# Synthesis of Novel 2,9-Disubstituted-6-morpholino Purine Derivatives Assisted by Virtual Screening and Modelling of Class I PI3K Isoforms

**DOI:** 10.3390/polym15071703

**Published:** 2023-03-29

**Authors:** Vítor Lobo, Ashly Rocha, Tarsila G. Castro, Maria Alice Carvalho

**Affiliations:** 1 Chemistry Centre, School of Sciences, University of Minho, 4710-057 Braga, Portugal; 2 CEB—Center of Biological Engineering, University of Minho, Campus de Gualtar, 4710-057 Braga, Portugal; 3 LABBELS—Associate Laboratory, Campus de Gualtar, 4710-057 Braga, Portugal

**Keywords:** cancer, isoform-specific PI3K inhibitors, docking, anticancer compounds, targeted therapy

## Abstract

The phosphatidylinositol-3 kinase (PI3K) pathway is one of the most frequently activated pathogenic signalling cascades in a wide variety of cancers. In the last 15 years, there has been an increase in the search for selective inhibitors of the four class I isoforms of PI3K, as they demonstrate better specificity and reduced toxicity in comparison to existing inhibitors. A ligand-based and target-based rational drug design strategy was employed to build a virtual library of 105 new compounds. Through this strategy, the four isoforms were compared regarding their activity pocket availability, amino acid sequences, and prone interactions. Additionally, a known active scaffold was used as a molecular base to design new derivatives. The virtual screening of the resultant library toward the four isoforms points to the obtention of 19 selective inhibitors for the PI3Kα and PI3Kγ targets. Three selective ligands, one for α-isoform and two for γ-isoform, present a ∆ (∆G_binding_) equal or greater than 1.5 Kcal/mol and were identified as the most promising candidates. A principal component analysis was used to establish correlations between the affinity data and some of the physicochemical and structural properties of the ligands. The binding modes and interactions established by the selective ligands in the active centre of the α and γ isoforms of PI3K were also investigated. After modelling studies, a synthetic approach to generate selective ligands was developed and applied in synthesising a set of derivatives that were obtained in good to excellent yield.

## 1. Introduction

One of the most common events in human cancer is the activation of the PI3K/AKT/mTOR signalling pathway. This is generally described as a consequence of genetic alterations, such as point mutations of PI3K genes or inactivation of PTEN. Thus, it presents a critical role in driving tumour initiation, progression, and cellular metabolism, associated with the mechanism of multiple drug resistance in a variety of cancers [1,2,3]. As such, that has prompted the development of inhibitors of several segments of this pathway, including the PI3K, AKT, and mTOR kinases [3,4,5].

Human cells express three different classes of PI3K enzymes [6,7]. These consist of eight PI3K isoforms divided into three classes based on structural, catalytic, and regulatory properties [8]. Among these kinases, the most widely studied is class I PI3Ks, which can be activated directly by cell surface receptors [9]. There are four members of the class I PI3Ks [5,10]. This class is subdivided into class IA (PI3Kα, β, and δ isoforms) and class IB (PI3Kγ isoform) [4]. The diversity in the ability of various PI3K isoforms to differentially sense signalling inputs is key to their broad and crucial physiological roles [11]. Class IA PI3Ks are activated by a variety of cell surface receptors, such as receptor tyrosine kinases (RTKs), G protein-coupled receptors (GPCRs), and the small G protein RAS, while class IB PI3K (the subunit PI3Kγ) is activated via the direct binding of p110γ to the Gβγ subunit of GPCRs [7,12,13]. The p110 catalytic subunit has five well-characterised domains: an *N*-terminal adaptor binding domain (ABD), a ras binding domain (RBD), a C2 domain, a helical domain, and a catalytic domain which is homologous to that of other lipid kinases [11].

PI3K has become an important anticancer drug target, and the development of PI3K inhibitors is of great interest to the pharmaceutical industry. Numerous PI3K inhibitors have exhibited good results in solid tumours in preclinical studies [9]. The use of these is currently considered a fruitful strategy to combat cancer, being mainly divided according to selectivity into three generic classes: pan-PI3K inhibitors, selective isoform PI3K inhibitors, and dual PI3K/mTOR inhibitors [3,14,15]. The development of specific PI3K isoform inhibitors aims to achieve the same goal as pan-PI3K inhibitors but without their adverse effects. This may offer a therapeutic benefit in a broad range of clinical settings for cancer and inflammatory and immunological diseases [16,17]. Selective inhibitors are therefore considered a new strategy to combat certain types of cancer depending on which isoform is amplified or mutated in the cancer [3].

Activating mutations in p110α are frequent in malignant tumours, with the gene encoding p110α, PIK3CA, being the second most frequent oncogene in human cancer [11]. PI3Kβ is another ubiquitously expressed class I PI3K. The knockout of PIK3CB avoids tumour formation in PTEN-null prostate cancer mouse models. PTEN loss or mutation is detected in a considerable fraction of tumours, including gliomas, breast, colon, lung, endometrial, and prostate cancers [18,19]. On the other hand, PI3Kγ is preferentially expressed in the hematopoietic system, specifically in leukocytes. It is a key regulator of cellular migration. Thus, various diseases related to an influx of inflammatory effector cells inhibitors could be treated by inhibiting this PI3K isoform, including inflammation, respiratory and metabolic disorders, and cancer [3]. Finally, hyperactivated PI3K signalling is common in leukaemia specimens and cultured cells [18]. Hence, targeting PI3Kδ may be beneficial both for autoimmune diseases and cancer. 

**Figure 1:**
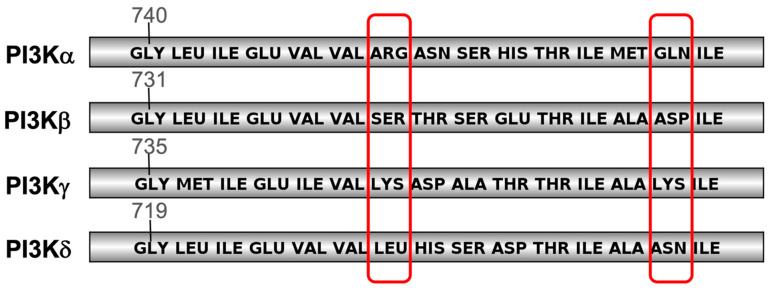
Binding pocket insights into PI3K isoforms. Sequence alignment indicates the conserved and variable residues in each PI3K isoform [21].

Isoform-specific PI3K inhibitors are expected to favour the safety inhibitory profile by reducing the toxicity and side-effects of current inhibitors [14,20]. Thus, understanding the molecular details of these four regulatory subunits can shed light on targeted therapy [11]. Figure 1 shows the sequence alignment of residues for the four class I PI3K isoforms. Given the high degree of similarity between the amino acid sequence forming the ATP-binding pockets of the four class I PI3Ks, it was expected that isoform-selective PI3K inhibitors would be difficult to obtain [13,21]. 

**Figure 2:**
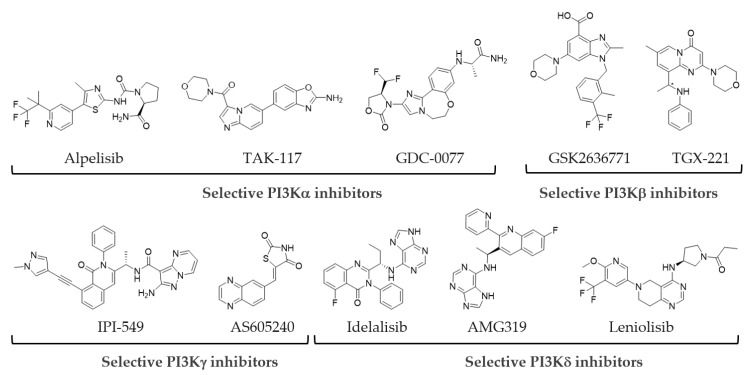
Examples of isoform-specific PI3K inhibitors approved for clinical use or under clinical development [19,20,21,22,23,24].

Class I PI3Ks’ ATP binding sites are highly homologous, differing only in a few residues divided into two regions [21]. Nonconserved residues in the two regions are highlighted between red rectangles in Figure 1 and can confer selectivity due to the chemical and conformational differences of these regions among the four isoforms. Of all these, the residues highlighted within the rectangles are the ones reported to be the most common residues that interact with selective inhibitors (Figure 2). Notably, they are different in each isoform. Hence, they can define specific interactions, allowing inhibitor specificity [21]. Despite the divergent roles of each isoform in different signalling contexts, tissues, and types of diseases have been extensively studied, continuous efforts are needed to explore the precise functions among PI3K isoforms to implement precise targeting [19]. 

**Figure 3:**
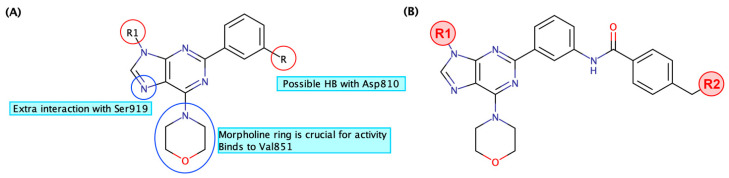
(**A**) Reported scaffold of the 2,9-disubstituted-6-morpholino purine derivatives and interaction-binding pattern with the active site of PI3Kα. (**B**) The base structure of the library of compounds designed in this work.

In the literature, a series of 2-9-disubstituted-6-morpholino purine derivatives are reported as potent and selective inhibitors of PI3Kα, with IC_50_ = 11 nM [3], while presenting some selectivity for PI3Kγ [22]. Figure 3A shows the scaffold and the binding interaction pattern of the constituent groups of this class of selective inhibitors with the active centre of PI3Kα [3,22]. Figure 3B displays the base structure that we used to design new derivatives. The reported ligands have at N9 the R^1^ = cyclohexylamine derivatives, a bulky hydrophobic group (Figure 3A). To explore the chemical space at N9, we introduced different groups as R^1^ (H, CH_3_, CH_2_Ph, Ar). Several works in the literature also mention that when R = nitrogen, the activity increases (Figure 3A). Given the space availability in the target, we kept R = nitrogen but added a planar aryl amide with different hydrophilic R^2^ groups (Figure 3B).

Herein, molecular docking was employed toward the four isoforms under study for a set of 105 molecules where R^1^ and R^2^ are varied to assist the design of new selective inhibitors. This large library of compounds aims to disclose structural patterns and physicochemical properties responsible for both activity and selectivity. Importantly, the structural differences among the four activity sites were also addressed using modelling techniques. Thus, a combined strategy, considering ligand-based and target-based properties, takes place to evolve the rational design of new anticancer compounds. In addition, we also report the synthetic approach that will allow the efficient synthesis of the compounds designed.

## 2. Methods

### 2.1. In Silico

#### 2.1.1. Class 1 Compounds

Due to the novelty of the molecules under study, their physicochemical properties were predicted and collected from Marvin Sketch 20.21 (ChemAxon; https://chemaxon.com/marvin (accessed on 15 December 2020). The partition coefficient (LogP), molecular weight (MW), and molar refractivity (Rf) were obtained directly from the software. To predict with some accuracy the charge, number of hydrogen bond acceptors (HBA), and number of hydrogen bond donors (HBD) of the ligands, the study was performed at physiological pH (7.4). When a ligand presented charge, the chosen structure was the most representative (the one with the highest percentage at the pH chosen).

Quantum chemical calculations, at the DFT level, were then used to prepare all the ligands for docking, aiming to obtain their optimised electronic structures. Calculations were conducted with the hybrid density functional B3LYP [23,24] together with the 6-31+G (d,p) basis set [25,26]. All molecules were computed with the Gaussian 09 suite of programs [27] in a vacuum and without vibrational corrections.

After obtaining the most stable/probable conformation, Open Babel [28] was used to transform Gaussian outputs into the PDBQT format, suitable to be used in AutoDock Vina [29].

#### 2.1.2. PI3K Isoforms

The PDB codes of the 4 PI3K isoforms are: 6PYS for PI3Kα [30], 4BFR for PI3Kβ [31], 6AUD for PI3Kγ [32], and 6TNR for PI3Kδ [33]. From the canonical amino acid sequence, obtained in FASTA format from the same database for each protein, the necessity of completing small gaps in each one to obtain the complete tertiary structure of the four targets was proved. Overall, the active site of the proteins was intact in all four isoforms, with only a few gaps in areas relatively far from the binding site, corresponding to more mobile regions, such as loops, turns, or bends, which should not interfere with the affinity result. In any case, the complete structures were those used as targets for the virtual screening.

For this purpose, SWISS-MODEL [34,35,36,37,38] was used as a homology-modelling tool to obtain the complete structures. From all the generated structures, in all four cases, the model chosen was the one with the highest GMQE (global model quality estimate). Notably, these structures were constructed upon their respective PDB code, maintaining a great alignment and conformational similarity to the experimental protein. Then, PyMOL [39] was used to prepare each protein for the subsequent methodology step. Firstly, all hydrogens were added to the respective macromolecules’ amino acids. Finally, the .pdb files were converted to .pdbqt in AutoDock Tools [40], resulting in a suitable format for the virtual screening. In this step, only the polar hydrogens are maintained.

#### 2.1.3. Virtual Screening

The affinity (∆G_binding_) of our ligands’ library was estimated by virtual screening against the 4 targets using AutoDock Vina [29]. The aim was to determine whether when molecules share a particular structural or physicochemical characteristic, the binding mode and energy will be similar for a specific isoform. To run the molecular docking experiments, the number of points and spacing of the grid box centre were determined. The centre was settled considering the special location of the original ligand found in each isoform. To assure that all the important residues at the active site were considered, the number of points was decided under the consideration that the final volume of the box could encompass both the ligand size and the amino acids from the active site. For this reason, for all the targets under study, the number of points in the dimension used for the grid box was 28 for all three dimensions, in a grid spacing of 1.0 Å [29]. An exhaustiveness of 20 and num_modes = 20 was used for each docking run. The grid box axial coordinates are summarised in the Supplementary Materials (Table S1).

#### 2.1.4. PCA

Principal component analysis (PCA) from SPSS software [41] was used to correlate the data listed for all the 105 reported ligands against the binding energy predicted for the α and γ isoforms of PI3K, forecasted by the docking experiments. This methodology allows the reduction of the dimensionality of data while increasing interpretability. It does so by creating new uncorrelated variables that successively maximise variance. This translates into finding new variables, principal components, which are linear functions of those in the original dataset, that successively maximise variance and that are uncorrelated with each other [42]. Hence, by using PCA, it is possible to, in a single graph, follow the relationship between the binding energy and all the properties of all the ligands, instead of using several linear correlation graphs (scatter plots) between only two variables that will not reveal some patterns as in PCA.

### 2.2. Chemical Synthesis

#### 2.2.1. General Information 

The 5-amino-4-cyanoformimidoylimidazoles **22a**–**h** used in this work were synthesised according to previously described procedures [43], compounds 23 were obtained following procedures reported in [44,45,46], and compounds **24**–**25** were synthesised according to procedures described in [47]. Solvents and other chemicals commercially available were used as shipped. The melting points (M.p., °C) were determined with a Gallenkamp melting point apparatus and are uncorrected. The reactions were monitored by thin layer chromatography (TLC) using Silica Gel 60 F254 (Merck, Darmstadt, Germany) with detection by UV light. ^1^H and ^13^C NMR spectra, including the ^1^H and ^13^C correlation spectra (HMQC and HMBC), were recorded on a Bruker Avance III NMR spectrometer at 400 and 100 MHz, respectively. Chemical shifts (*δ*) were reported in parts per million (ppm), and the coupling constants, *J*, were reported in hertz (Hz). Infrared (IR) spectra were recorded with a FT-IR Bomem MB 104 using nujol mulls and NaCl cells. Elemental analyses were performed with a LECO CHNS-932 instrument.

#### 2.2.2. General Procedure for the Synthesis of Compounds **24**

The nitrobenzalaldehyde (1.1 equiv) and Et_3_N (10 equiv.) were added to a suspension of 5-amino-4-amidino-imidazoles **23** in DMSO. The reaction was carried out at 80 °C under efficient magnetic stirring and was carefully monitored by TLC. When the starting reagent was absent, the reaction was cooled to room temperature, and distilled water was added to the suspension. The solid was filtered and washed with distilled water followed by cold ethanol and cold diethyl ether and identified as compound **24**. Analytical pure samples were obtained by recrystallisation of the isolated solids from ethanol.

##### 4-(2-(3-Nitrophenyl)-9-(3,4-dimethylphenyl)-9H-purin-6-yl)morpholine (**24e**) 

Compound **24e** (1.92 g, 4.46 mmol, 75%) was obtained as a light-yellow solid from the reaction of compound **23e** (1.77 g, 5.92 mmol) with 3-nitrobenzaldehyde (0.98 g, 6.52 mmol) and Et_3_N (8.26 mL, 59.20 mmol) in DMSO (3 mL) after 21.5 h. M.p. 227–229 °C. IR (nujol) νmax: 3102, 1594, 1573, 1530, 1504 cm^−1^. ^1^H NMR (400 MHz, DMSO-*d*_6_) *δ*: 9.06 (t, 1H, *J*^4^ = 2.0 Hz), 8.72 (dt, 1H, *J*^3^ = 8.0 Hz, *J*^4^ = 2.0 Hz), 8.48 (s, 1H), 8.24 (ddd, 1H, *J*^3^ = 8.0 Hz, *J*^4^ = 2.0, *J*^4^ = 0.8 Hz), 7.75 (t, 1H, *J*^3^ = 8.0 Hz), 7.72 (s, 1H), 7.62 (dd, 1H, *J*^3^ = 8.4 Hz, *J*^4^ = 2.0 Hz), 7.37 (d, 1H, *J*^3^ = 8.4 Hz), 4.36 (t, 4H, *J*^3^ = 4.8 Hz), 3.83 (t, 4H, *J*^3^ = 4.8 Hz), 2.38 (s, 3H), 2.35 (s, 3H). ^13^C NMR (100 MHz, DMSO-*d*_6_) *δ*: 155.01, 153.15, 150.94, 147.96, 139.70, 139.22, 137.07, 135.52, 133.14, 132.19, 129.80, 129.32, 123.87, 123.51, 121.42, 120.19, 118.91, 65.71, 45.08, 18.68, 18.18. MS (ESI) 452 [M^+^ − 1 + Na]. HRMS (ESI): *m*/*z* [M^+^ + 1] calcd for C_23_H_23_N_6_O_3_: 431.1832; found: 431.1837.

##### 4-(2-(3-Nitrophenyl)-9-(4-aniline)-9H-purin-6-yl)morpholine (**24h**)

Compound **24h** (1.72 g, 4.13 mmol, 79%) was obtained as a yellow solid from the reaction of compound **23h** (1.50 g, 5.25 mmol) with 3-nitrobenzaldehyde (0.88 g, 5.77 mmol) and Et_3_N (7.31 mL, 52.46 mmol) in DMSO (3.5 mL) after 16 h. M.p. 218–220 °C. IR (nujol) νmax: 3435, 3341, 3100, 1593, 1572, 1523 cm^−1^. ^1^H NMR (400 MHz, DMSO-*d*_6_) *δ*: 8.97 (t, 1H, *J*^4^ = 2.0 Hz), 8.68 (dd, 1H, *J*^3^ = 8.0 Hz, *J*^4^ = 2.0 Hz), 8.40 (s, 1H), 8.25 (ddd, 1H, *J*^3^ = 8.0 Hz, *J*^4^ = 2.0 Hz, *J*^4^ = 0.8 Hz), 7.71 (t, 1H, *J*^3^ = 8.0 Hz), 7.43 (d, 2H, *J*^3^ = 8.4 Hz), 6.76 (d, 2H, *J*^3^ = 8.4 Hz), 5.43 (s, 2H, NH_2_, exchangeable by D_2_O), 4.30 (br.s, 4H,), 3.77 (t, 4H, *J*^3^ = 4.8 Hz). ^13^C NMR (100 MHz, DMSO-*d*_6_) *δ*: 154.84, 153.11, 151.19, 148.75, 147.98, 140.21, 139.82, 133.72, 129.90, 124.97, 124.18, 123.10, 121.70, 118.85, 113.88, 66.21, 45.34. MS (ESI) 418 [M^+^ + 1]. HRMS (ESI): *m*/*z* [M^+^ + 1] calcd for C_21_H_20_N_7_O_3_: 418.1628; found: 418.1622.

##### *N*-(4-(6-Morpholino-2-(3-nitrophenyl)-9H-purin-9-yl)phenyl)benzamide (**24i**) 

Compound **24h** (0.74 g, 1.77 mmol), benzoic anhydride (1.7 eq., 0.70 g, 3.07 mmol), Et_3_N (1.7 eq., 0.43 mL, 3.07 mmol), and DMSO (1.5 mL) were mixed in a vial. The vial was closed, and the reaction was carried out at 80 °C under efficient magnetic stirring and was carefully monitored by TLC. After 3 h and 30 min, the absence of the starting reagent **24h** was confirmed. The reaction was cooled down to room temperature. Then, acetonitrile and distilled water were added to the solution until a grey solid precipitated. The resulting suspension was cooled down in an ice bath, and the solid was filtered off and washed successively with water, cold acetonitrile, and diethyl ether. The resulting off-white solid was identified as **24i** (0.92 g, 1.76 mmol, 99%). M.p. 266–268 °C. IR (nujol) νmax: 3427, 3286, 1665, 1648, 1591, 1569, 1521 cm^−1^. ^1^H NMR (400 MHz, DMSO-*d*_6_) δ: 10.49 (s, 1H, NH, exchangeable by D_2_O), 8.99 (t, 1H, *J^4^* =2.4 Hz,), 8.71 (dd, 1H, *J*^3^ = 8.0 Hz, *J*^4^ = 2.4 Hz), 8.58 (s, 1H), 8.25 (ddd, 1H, *J*^3^ = 8.0 Hz, *J*^4^ = 2.4 Hz, *J*^4^ = 0.8 Hz), 8.02–7.97 (m, 4H), 7.73 (t, 1H, *J*^3^ = 8.0 Hz), 7.63–7.52 (m, 3H), 7.28 (d, 2H, *J*^3^ = 8.0 Hz), 4.32 (br s, 4H), 3.78 (t, 4H, *J*^3^ = 4.8 Hz). ^13^C NMR (100 MHz, DMSO-*d*_6_) δ: 166.07, 155.42, 153.38, 151.27, 148.23, 140.00, 139.85, 138.84, 134.82, 134.05, 132.02, 130.35, 130.24, 128.70, 127.89, 124.58, 124.05, 121.96, 121.25, 119.18, 66.40, 45.44. MS (ESI) 522 [M^+^ + 1]. HRMS (ESI): *m*/*z* [M^+^ + 1] calcd for C_28_H_24_N_7_O_4_: 522.1890; found: 522.1893.

#### 2.2.3. General Procedure for the Reduction of 2-(3-Nitrophenyl)-purine Derivatives (**24**)

2-nitrophenyl-purine derivatives **24** were combined, in a round bottom flask, with iron powder (8 eq.) and acetic acid (20 eq.) in a 95% ethanol solution. The reaction mixture, under nitrogen atmosphere, was submitted to reflux, and the reaction was monitored by TLC (ethyl acetate). When the TLC showed absence of starting material, the suspension was cooled to approximately 20 °C. A 25% aqueous ammonia solution was added to the suspension until pH = 10–11. The suspension was kept at room temperature for 12–18 h. The residue in suspension was filtered through a diatomaceous earth column (2–3 cm). The solution was concentrated using a rotary evaporator until total removal of ethanol. The resulting suspension was cooled using an ice bath, and the solid was filtered, washed successively with water and diethyl ether, and identified as compound **25**. Analytical pure samples were obtained either by flash chromatography using THF as solvent or by recrystallisation of the solids from dichloromethane.

##### 3-(9-(3,4-Dimethylphenyl)-6-morpholino-9H-purin-2-yl)aniline (**25e**) 

Compound **25e** (1.13 g, 2.82 mmol, 86%) was obtained as a white solid from the reaction of compound **24e** (1.42 g, 3.31 mmol) with iron powder (1.48 g, 26.50 mmol) and acetic acid (3.79 mL, 66.20 mmol), in 150 mL of ethanol (95%), after 6 h at 80 °C and 17 h at room temperature. M.p. 163–165 °C. IR (nujol) νmax: 3433, 3354, 3239, 3098, 1636, 1577, 1515 cm^−1^. ^1^H NMR (400 MHz, DMSO-*d*_6_) δ: 8.50 (s, 1H), 7.64 (m, 2H), 7.59 (t, 1H, *J*^4^ = 1.6 Hz), 7.52 (d, 1H, *J*^3^ = 8.0 Hz), 7.36 (d, 1H, *J*^3^ = 8.0 Hz), 7.08 (t, 1H, *J*^3^ = 8.0 Hz), 6.63 (dd, 1H, *J*^3^ = 8.0 Hz, *J*^4^ = 1.6 Hz), 5.13 (s, 2H, NH_2_, exchangeable by D_2_O), 4.32 (br s, 4H), 3.78 (t, 4H, *J*^3^ = 4.8 Hz), 2.33 (s, 3H), 2.30 (s, 3H). ^13^C NMR (100 MHz, DMSO-*d*_6_) δ: 158.17, 153.12, 151.46, 148.52, 139.24, 138.79, 137.61, 135.79, 132.79, 130.27, 128.60, 124.21, 120.78, 118.50, 115.80, 115.56, 113.44, 66.26, 45.20, 19.52, 18.97. MS (ESI) 401 [M^+^ + 1]. HRMS (ESI): *m*/*z* [M^+^ + 1] calcd for C_23_H_25_N_6_O: 401.2090; found: 401.2093.

##### *N*-(4-(2-(3-Aminophenyl)-6-morpholino-9H-purin-9-yl)phenyl)benzamide (**25h**) 

Compound **25h** (0.51 g, 1.04 mmol, 97%) was obtained as an off-white solid from the reaction of compound **24i** (0.56 g, 1.06 mmol) with iron powder (0.48 g, 8.52 mmol) and acetic acid (1.22 mL, 21.29 mmol), in 150 mL of ethanol (95%), after 7 h. M.p. 258–260 °C. IR (nujol) νmax: 3458, 3324, 3133, 1651, 1584, 1531 cm^−1^. ^1^H NMR (400 MHz, DMSO-*d*_6_) δ: 10.50 (s, 1H, NH, exchangeable by D_2_O), 8.46 (s, 1H), 7.93 (m, 4H), 7.85 (d, 2H, *J*^3^ = 9.2 Hz), 7.58 (m, 2H), 7.52 (m, 3H), 7.09 (t, 1H, *J*^3^ = 8.0 Hz), 6.64 (ddd, 1H, *J*^3^ = 8.0 Hz, *J*^4^ = 2.4, *J*^4^ = 0.8 Hz), 4.28 (br.s, 4H), 3.75 (t, 4H, *J*^3^ = 4.8 Hz). ^13^C NMR (100 MHz, DMSO-*d*_6_) δ: 166.84, 158.89, 153.74, 152.04, 148.82, 139.76, 139.27, 138.82, 135.00, 132.59, 131.14, 129.45, 129.23, 128.21, 124.47, 122.03, 118.89, 116.84, 116.61, 114.21, 66.86, 45.81. MS (ESI) 492 [M^+^ + 1]. HRMS (ESI): *m*/*z* [M^+^ + 1] calcd for C_28_H_26_N_7_O_2_: 492.2148; found: 492.2146. 

#### 2.2.4. General Procedure for the Acylation of 2-(3-Aminophenyl)-purine Derivatives **25**

Under anhydrous conditions, the 2-(3-aminophenyl)-purine derivatives **25** were solubilised in dry THF. To the respective solutions, K_2_CO_3_ (1.1–1.5 eq.) and 4-(chloromethyl)benzoyl chloride (1.05–1.30 eq.) were added. The suspension was left to react in a closed vial, at room temperature, under efficient magnetic stirring, and was carefully monitored by TLC. When the absence of limiting reagent was confirmed, water was added to the reaction mixture. The resulting suspension was cooled using an ice bath, and the solid was filtered and then washed with water, cold ethanol, and diethyl ether. The solid obtained was isolated and identified as the pure desired product.

##### Synthesis of 4-(Chloromethyl)-*N*-(3-(6-morpholino-9H-purin-2-yl)phenyl)benzamide (**26a**) 

Compound **26a** (0.21 g, 0.47 mmol, 74%) was obtained as a beige solid from the reaction of compound **25a** (0.12 g, 0.40 mmol) with 4-(chloromethyl)benzoyl chloride (0.79 g, 0.42 mmol, 1.05 eq.) in 4 mL of dry THF after 20 min. M.p. > 300 °C. IR (nujol) νmax: 3287, 3105, 2961, 2856, 1647, 1610, 1572, 1538 cm^−1^. ^1^H NMR (400 MHz, DMSO-*d*_6_) *δ*: 13.14 (br s, 1H, NH exchangeable by D_2_O), 10.40 (s, 1H, NH, exchangeable by D_2_O), 8.75 (t, 1H, *J*^3^ = 8.0 Hz), 8.16 (s, 1H), 8.11 (d, 1H, *J*^3^ = 8.0 Hz), 7.99 (d, 2H, *J*^3^ = 8.0 Hz), 7.88 (d, 1H, *J*^3^ = 8.8 Hz), 7.59 (d, 2H, *J*^3^ = 8.0 Hz), 7.43 (t, 1H, *J*^3^ = 8.0 Hz), 4.84 (s, 2H), 4.31 (br s, 4H), 3.77 (t, 4H, *J*^3^ = 4.0 Hz) ppm. ^13^C NMR (100 MHz, DMSO-*d*_6_) *δ*: 165.2, 156.8, 153.0, 152.6, 141.0, 139.1, 139.0, 138.9, 134.9, 128.8, 128.5, 123.2, 121.7, 120.0, 118.0. 66.3, 45.5, 45.2 ppm. MS (ESI) 450 [M^+^ + 1]. Anal. calcd for C_23_H_21_ClN_6_O_2_. 0.2 H_2_O: C, 60.33; H, 4.81; N, 18.27. found: C, 60.21; H, 4.85; N, 18.32.

##### 4-(Chloromethyl)-*N*-(3-(9-methyl-6-morpholino-9H-purin-2-yl)phenyl)benzamide (**26b**)

Compound **26b** (0.27 g, 0.58 mmol, 81%) was obtained as an ivory solid from the reaction of compound **25b** (0.22 g, 0.71 mmol) with 4-(chloromethyl)benzoyl chloride (0.141 g, 0.75 mmol, 1.05 eq.) and K_2_CO_3_ (0.108 g, 0.44 mmol, 1.1 eq.), in 5 mL of dry THF, after 2 h and 10 min. M.p. 224–226 °C. IR (nujol) νmax: 3301, 2863, 1644, 1608, 1570, 1532, 1507 cm^−1^. ^1^H NMR (400 MHz, DMSO-*d*_6_) *δ*: 10.42 (s, 1H, NH, exchangeable by D_2_O), 8.70 (t, 1H, *J*^4^ = 1.6 Hz), 8.16 (s, 1H), 8.15 (t, 1H, *J*^4^ = 1.2 Hz), 7.99 (d, 2H, *J*^3^ = 8.0 Hz), 7.94 (dd, 1H, *J*^4^ = 2.0 Hz, *J*^3^ = 8.0 Hz), 7.59 (d, 2H, *J*^3^ = 8.0 Hz), 7.44 (t, 1H, *J*^3^ = 8.0 Hz), 4.84 (s, 2H), 4.30 (br s, 4H), 3.77 (s, 3H), 3.42 (t, 4H, *J*^3^ = 4.4 Hz) ppm. ^13^C NMR (100 MHz, DMSO-*d*_6_) *δ*: 165.3, 156.7, 153.0, 152.1, 141.3, 141.1, 139.2, 139.0, 134.9, 128.8, 128.5, 128.2, 123.4, 121.9, 120.0, 118.2, 66.3, 45.5, 45.3, 29.5 ppm. MS (ESI) 464 [M^+^ + 1]. Anal. calcd for C_24_H_23_ClN_6_O_2_. 0.3 H_2_O: C, 61.55; H, 5.08; N, 17.94. Found: C, 61.33; H, 4.88; N, 17.93.

##### *N*-(3-(9-Benzyl-6-morpholino-9H-purin-2-yl)phenyl)-4-(chloromethyl)benzamide (**26c**) 

Compound **26c** (0.22 g, 0.42 mmol, 69%)) was obtained as a beige solid from the reaction of compound **25c** (0.25 g, 0.61 mmol) with 4-(chloromethyl)benzoyl chloride (0.121 g, 0.64 mmol, 1.05 eq.) and K_2_CO_3_ (0.093 g, 0.67 mmol, 1.1 eq.), in 4 mL of dry THF, after 1 h and 30 min. M.p. 254–256 °C. IR (nujol) νmax: 3301, 3073, 2857, 1645, 1609, 1575, 1536 cm^−1^. ^1^H NMR (400 MHz, DMSO-*d*_6_) *δ*: 10.41 (s, 1H, NH, exchangeable by D_2_O), 8.74 (br s, 1H), 8.33 (s, 1H), 8.16 (d, 1H, *J*^3^ = 7.6 Hz), 7.99 (d, 2H, *J*^3^ = 8.0 Hz), 7.94 (d, 1H, *J*^3^ = 7.6 Hz), 7.59 (d, 2H, *J*^3^ = 8.0 Hz), 7.47–7.41 (m, 4H), 7.34 (t, 2H, *J*^3^ = 7.2 Hz), 7.27 (t, 2H, *J*^3^ = 7.2 Hz), 5.47 (s, 2H), 4.84 (s, 2H), 4.31 (br s, 4H), 3.77 (t, 4H, *J*^3^ = 4.0 Hz) ppm; ^13^C NMR (100 MHz, DMSO-*d*_6_) *δ*: 165.3,156.9, 153.0, 151.7, 141.0, 140.5, 139.2, 138.8, 137.1, 134.9, 128.8, 128.7, 128.5, 128.1, 127.9, 127.8, 123.3, 121.9, 120.0, 118.2, 66.3, 46.2, 45.5, 45.2 ppm. MS (ESI) 540 [M^+^ + 1]. Anal. calcd for C_30_H_27_ClN_6_O_2_. 0.6 H_2_O: C, 65.53; H, 5.17; N, 15.28. Found: C, 65.79; H, 5.43; N, 14.92.

##### 4-(Chloromethyl)-*N*-(3-(6-morpholino-9-phenyl-9H-purin-2-yl)phenyl)benzamide (**26d**) 

Compound **26d** (0.31 g, 0.59 mmol, 95%) was obtained as a light-beige solid from the reaction of compound **25d** (0.24 g, 0.62 mmol) with 4-(chloromethyl)benzoyl chloride (0.123 g, 0.65 mmol, 1.05 eq.) and K_2_CO_3_ (0.094 g, 0.68 mmol, 1.1 eq.), in 3.3 mL of dry THF, after 1 h and 25 min. M.p. 220–222 °C. IR (nujol) νmax: 3301, 2962, 2855, 1652, 1575, 1533 cm^−1^. ^1^H NMR (400 MHz, DMSO-*d*_6_) *δ*: 10.41 (s, 1H, NH, exchangeable by D_2_O), 8.66 (s, 1H), 8.62 (s, 1H), 8.11 (d, 2H, *J*^3^ = 8.0 Hz), 7.98–7.97 (m, 4H), 7.66–7.58 (m, 4H), 7.50–7.42 (m, 2H), 4.84 (s, 2H), 4.37 (br s, 4H), 3.80 (t, 4H, *J*^3^ = 4.4 Hz) ppm; ^13^C NMR (100 MHz, DMSO-*d*_6_) *δ*: 165.3, 157.4, 153.2, 151.4, 141.0, 139.5, 139.1, 138.6, 135.0, 134.9, 129.6, 128.8, 128.5, 128.1, 127.6, 123.3, 123.3, 122.0, 119.9, 118.8, 66.3, 45.4, 45.2 ppm. MS (ESI) 526 [M^+^ + 1]. Anal. calcd for C_29_H_25_ClN_6_O_2_. 0.4 H_2_O: C, 65.20; H, 5.02; N, 15.73. Found: C, 65.37; H, 5.07 N, 15.32.

##### 4-(Chloromethyl)-*N*-(3-(9-(3,4-dimethylphenyl)-6-morpholino-9H-purin-2-yl)phenyl)benzamide (**26e**) 

Compound **26e** (1.08 g, 1.96 mmol, 97%) was obtained as an off-white solid from the reaction of compound **25e** (0.80 g, 2.01 mmol) with 4-(chloromethyl)benzoyl chloride (0.47 g, 2.51 mmol, 1.25 eq.) and K_2_CO_3_ (0.35 g, 2.51 mmol, 1.25 eq.), in 10 mL of dry THF, after 2.5 h. M.p. 228–230 °C. IR (nujol) νmax: 3282, 1645, 1609, 1575, 1533, 1508 cm^−1^. ^1^H NMR (400 MHz, DMSO-*d*_6_) *δ*: 10.41 (s, 1H, NH, exchangeable by D_2_O), 8.71 (t, 1H, *J*^4^ = 2.0 Hz), 8.54 (s, 1H), 8.09 (dt, 1H, *J*^3^ = 8.0 Hz, *J*^4^ = 0.8 Hz), 7.96 (d, 2H, *J*^3^ = 8.0 Hz), 7.90 (ddd, 1H, *J*^3^ = 8.0 Hz, *J*^4^ = 2.0 Hz, *J*^4^ = 0.8 Hz), 7.73 (d, 1H, *J*^4^ = 2.0 Hz), 7.64 (dd, 1H, *J*^3^ = 8.0 Hz, *J*^4^ = 2.0 Hz), 7.58 (d, 2H, *J*^3^ = 8.0 Hz), 7.44 (t, 1H, *J*^3^ = 8.0 Hz), 7.36 (d, 1H, *J*^3^ = 8.0 Hz), 4.84 (s, 2H), 4.35 (br s, 4H), 3.79 (t, 4H, *J*^3^ = 4.8 Hz), 2.35 (s, 3H), 2.30 (s, 3H). ^13^C NMR (100 MHz, DMSO-*d*_6_) *δ*: 165.34, 157.37, 153.27, 151.46, 141.10, 139.54, 139.18, 138.73, 137.80, 135.99, 134.91, 132.79, 130.39, 128.86, 128.58, 128.16, 124.28, 123.36, 122.07, 120.73, 120.04, 118.77, 66.33, 45.50 (2C), 19.55, 19.05. MS (ESI) 553 [M^+^]. HRMS (ESI): *m*/*z* [M^+^] calcd for C_31_H_29_ClN_6_O_2_: 552.2041; found: 552.2047. 

##### 4-(Chloromethyl)-*N*-(3-(9-(3-fluorophenyl)-6-morpholino-9H-purin-2-yl)phenyl)benzamide (**26f**)

Compound **26f** (0.25 g, 0.46 mmol, 86%) was obtained as an off-white solid from the reaction of compound **25f** (0.21 g, 0.53 mmol) with 4-(chloromethyl)benzoyl chloride (0.105 g, 0.56 mmol, 1.05 eq.) and K_2_CO_3_ (0.081 g, 0.58 mmol, 1.1 eq.), in 5 mL of dry THF, after 1 h and 10 min.. M.p. 252–254 °C. IR (nujol) νmax: 3278, 3083, 2963, 2855, 1651, 1599, 1567, 1534 cm^−1^. ^1^H NMR (400 MHz, DMSO-*d*_6_) *δ*: 10.41 (s, 1H, NH, exchangeable by D_2_O), 8.69 (t, 2H, *J*^4^ = 2.0 Hz), 8.11 (dt, 1H, *J*^3^ = 8.0 Hz, *J*^4^ = 1.2 Hz), 7.99-7.93 (m, 5H), 7.68 (dt, 1H, *J*^3^ = 8.0 Hz, *J*^4^ = 6.4 Hz), 7.60 (d, 2H, *J*^3^ = 8.8Hz), 7.46 (t, 1H, *J*^3^ = 8.0 Hz), 7.33 (tdd, 1H, *J*^3^ = 8.4 Hz, *J*^4^ = 2.4 Hz, *J*^4^ = 0.8 Hz), 4.85 (s, 2H), 4.36 (br s, 4H), 3.80 (t, 4H, *J*^3^ = 4.8 Hz) ppm; ^13^C NMR (100 MHz, DMSO-*d*_6_) *δ*: 165.3, 162.3 (d, *J*^2^ = 243.0 Hz), 157.5, 153.2, 151.3, 141.03, 139.3, 139.2, 138.5, 136.5 (d, *J*^3^ = 10.0 Hz), 134.9, 131.3 (d, *J*^3^ = 10.0 Hz), 128.8, 128.6, 128.1, 123.3, 122.1, 120.0, 119.0 (d, *J*^4^ = 3.0 Hz), 118.8, 114.3 (d, *J*^2^ = 20.0 Hz), 110.2 (d, *J*^2^ = 26.0 Hz), 66.2, 45.44, 45.2 ppm. MS (ESI) 543 [M^+^]. Anal. calcd for C_29_H_24_FClN_6_O_2_. 0.35 H_2_O: C, 63.41; H, 4.53; N, 15.30. Found: C, 63.35; H, 4.83; N, 15.57.

##### 4-(Chloromethyl)-*N*-(3-(9-(4-chlorophenyl)-6-morpholino-9H-purin-2-yl)phenyl)benzamide (**26g**) 

Compound **26g** (0.33 g, 0.63 mmol, 95%) was obtained as a light-yellow solid from the reaction of compound **25g** (0.25 g, 0.61 mmol) with 4-(chloromethyl)benzoyl chloride (0.121 g, 0.64 mmol, 1.05 eq.) and K_2_CO_3_ (0.093 g, 0.67 mmol, 1.1 eq.), in 5 mL of dry THF, after 1 h and 10 min.. M.p. 261-263 °C. IR (nujol) νmax: 3302, 2959, 2857, 1646, 1598, 1574, 1530, 1511 cm^−1^. ^1^H NMR (400 MHz, DMSO-*d*_6_) δ: 10.40 (s, 1H, NH, exchangeable by D_2_O), 8.65 (t, 1H, *J*^4^ = 2.0 Hz), 8.63 (s, 1H), 8.11 (dt, 1H, *J*^3^ = 8.0 Hz, *J*^4^ = 2.0 Hz), 8.02 (d, 2H, *J*^3^ = 8.8 Hz), 7.98-7.96 (m, 3H), 7.70 (d, 2H, *J*^3^ = 8.8 Hz), 7.59 (d, 2H, *J*^3^ = 8.0 Hz), 7.44 (t, 1H, 4.84 (s, 2H), 4.35 (br s, 4H), 3.79 (t, 4H, *J*^3^ = 4.8 Hz) ppm; ^13^C NMR (100 MHz, DMSO-*d*_6_) δ: 165.39, 157.5, 153.3, 151.4, 141.1, 139.3, 138.6, 134.9, 134.0, 132.0, 129.6, 128.9, 128.2, 127.7, 125.1, 123.4, 122.1, 120.0, 118.7, 66.31, 45.5, 45.31 ppm. MS (ESI) 559 [M^+^]. Anal. calcd for C_29_H_24_Cl_2_N_6_O_2_. 0.75 H_2_O: C, 60.87; H, 4.41; N, 14.61. Found: C, 60.79; H, 4.49; N, 14.67.

##### *N*-(3-(9-(4-Benzamidophenyl)-6-morpholino-9H-purin-2-yl)phenyl)-4-(chloromethyl)benzamide (**26h**)

Compound **26h** (0.21 g, 0.33 mmol, 98%) was obtained as a light-brown solid from the reaction of compound **25h** (0.17 g, 0.34 mmol) with 4-(chloromethyl)benzoyl chloride (0.09 g, 0.45 mmol, 1.3 eq.) and K_2_CO_3_ (0.06 g, 0.45 mmol, 1.3 eq.), in 6 mL of dry THF, after 1 h. M.p. 236–238 °C. IR (nujol) νmax: 3417, 1782, 1650, 1606, 1578, 1527 cm^−1^. ^1^H NMR (400 MHz, DMSO-*d*_6_) δ: 10.49 (s, 1H, NH, exchangeable by D_2_O), 10.43 (s, 1H, NH, exchangeable by D_2_O), 8.65 (t, 1H, *J*^4^ = 1.6 Hz), 8.59 (s, 1H), 8.13 (d, 1H, *J*^3^ = 8.0 Hz), 8.05–7.92 (m, 9H), 7.63–7.53 (m, 5H), 7.45 (t, 1H, *J*^3^ = 8.0 Hz), 4.83 (s, 2H), 4.36 (br s, 4H), 3.80 (t, 4H, *J*^3^ = 4.8 Hz). ^13^C NMR (100 MHz, DMSO-*d*_6_) δ: 165.81, 165.38, 157.43, 153.25, 151.46, 141.05, 139.48, 139.16, 138.71, 138.59, 134.75, 134.94, 131.79, 130.47, 128.81, 128.57, 128.49, 128.16, 127.77, 123.78, 123.44, 122.09, 121.12, 119.99, 118.72, 66.32, 45.48, 45.21. MS (ESI) 644 [M^+^]. HRMS (ESI): *m*/*z* [M^+^] calcd for C_36_H_30_ClN_7_O_3_: 643.2099; found: 643.2095

#### 2.2.5. Synthesis of *N*-(3-(9-(3,4-Dimethylphenyl)-6-morpholino-9H-purin-2-yl)phenyl)-4-((1,3-dioxoisoindolin-2-yl)methyl)benzamide (**27**) 

A mixture of **26e** (0.19 g, 0.37 mmol) and potassium phthalimide (1.2 eq., 81.6 mg, 0.44 mmol) was suspended in DMSO (1.5 mL), and Et_3_N (1.2 eq., 62 µL, 0.44 mmol) was added. The reaction was carried out in a closed vial at 100 °C under efficient magnetic stirring and was carefully monitored by TLC. After 1.5 h, the absence of the starting material was confirmed. The reaction was cooled down to room temperature. Then, distilled water was added to the suspension and cooled down in an ice bath. The solid was filtered off and washed successively with water, cold ethanol, and diethyl ether. The resulting white solid was identified as **27** (0.22 g, 0.35 mmol, 94%). M.p. > 280 °C. IR (nujol) νmax: 3292, 1710, 1646, 1608, 1569, 1533, 1514 cm^−1^. ^1^H NMR (400 MHz, DMSO-*d*_6_) *δ*: 10.34 (s, 1H, NH, exchangeable by D_2_O), 8.68 (t, 1H, *J*^4^ = 1.6 Hz), 8.54 (s, 1H), 8.08 (dt, 1H, *J*^3^ = 8.0 Hz, *J*^4^ = 1.6 Hz), 7.92–7.85 (m, 7H), 7.73 (d, 1H, *J*^4^ = 2.0 Hz), 7.64 (dd, 1H, *J*^3^ = 8.0 Hz, *J*^4^ = 2.0 Hz), 7.45 (d, 2H, *J*^3^ = 8.4 Hz), 7.42 (t, 1H, *J*^3^ = 8.0 Hz), 7.36 (d, 1H, *J*^3^ = 8.0 Hz), 4.86 (s, 2H), 4.34 (br s, 4H), 3.78 (t, 4H, *J*^3^ = 4.8 Hz), 2.34 (s, 3H,), 2.29 (s, 3H). ^13^C NMR (100 MHz, DMSO-*d*_6_) *δ*: 167.78, 165.45, 157.34, 153.24, 151.44, 140.21, 139.52, 139.19, 138.68, 137.78, 135.97, 134.69, 134.23, 132.77, 131.61, 130.36, 128.54, 128.10, 127.28, 124.26, 123.34, 123.27, 122.02, 120.70, 120.00, 118.75, 66.31, 45.48, 40.70, 19.53, 19.03. MS (ESI) 663 [M^+^]. HRMS (ESI): *m*/*z* [M^+^] calcd for C_39_H_33_N_7_O_4_: 663.2594; found: 663.2591. 

#### 2.2.6. General Procedure for the Reaction of Compounds **26** with Nitrogen Nucleophiles

A nitrogen nucleophile (5 eq.) was added to a suspension of acylated 2-(3-aminophenyl)-purine derivatives **26** in dioxane. The mixture was stirred in a closed vial at 110 °C until all the starting material was consumed (evidenced by TLC). Then, the reaction was allowed to cool down to room temperature, and distilled water was added. The resulting suspension was cooled down in an ice bath, and the solid was filtered off and washed successively with water, cold ethanol, and cold diethyl ether.

##### 4-((4-Methylpiperazin-1-yl)methyl)-*N*-(3-(6-morpholino-9H-purin-2-yl)phenyl)benzamide (**1b**)

Compound **1b** (0.06 g, 0.12 mmol, 63%) was obtained as a beige solid from the reaction of **26a** (0.09 g, 0.19 mmol) with *N*-methyl-piperazine (67 μL, 0.6 mmol, 5 eq.) in dioxane (0.5 mL) after 2 h and 20 min. M.p. 273–275 °C. IR (nujol) νmax: 3279, 2793, 1648, 1575, 1532 cm^−1^. ^1^H NMR (400 MHz, DMSO-*d*_6_): *δ* 13.06 (br s, 1H, NH, exchangeable by D_2_O), 10.33 (s, 1H, NH, exchangeable by D_2_O), 8.75 (t, 1H, *J^4^* =1.6 Hz), 8.16 (s, 1H), 8.10 (dt, 1H, *J*^3^ = 7.6 Hz, *J*^4^ = 0.8 Hz), 7.94 (d, 2H, *J*^3^ = 8.4 Hz), 7.87 (d, 1H, *J*^3^ = 8.0 Hz), 7.44 (d, 2H, *J*^3^ = 8.4 Hz), 7.42 (t, 1H, *J*^3^ = 8.0 Hz), 4.31 (br s, 4H), 3.77 (t, 4H, *J*^3^ = 4.8 Hz), 3.55 (s, 2H), 2.39 (m, 8H), 2.19 (s, 3H) ppm; ^13^C NMR (100 MHz, DMSO-*d*_6_): *δ* 165.5, 156.8, 152.9, 152.6, 142.12, 139.23, 138.96, 133.7, 128.7, 128.4, 127.7, 123.0, 121.7, 119.8, 118.01, 113.9, 66.3, 61.5, 54.6, 52.34, 45.7, 45.5, 43.06 ppm. MS (ESI) 513 [M^+^]. Anal. calcd for C_28_H_32_N_8_O_2_. 0.1 H_2_O: C, 65.38; H, 6.31; N, 21.78. Found: C, 65.27; H, 6.35; N, 21.55.

##### *N*-(3-(9-Methyl-6-morpholino-9H-purin-2-yl)phenyl)-4-((4-methylpiperazin-1-yl)methyl)benzamide (**2b**)

Compound **2b** (0.13 g, 0.24 mmol, 63%) was obtained as a white solid from the reaction of **26b** (0.18 g, 0.38 mmol) with *N*-methyl-piperazine (134 μL, 1.2 mmol, 5 eq.) in dioxane (0.5 mL) after 12 h. M.p. 215–217 °C. IR (nujol) νmax: 3325, 2951, 2838, 2790, 1651, 1608, 1579, 1533 cm^−1^. ^1^H NMR (400 MHz, DMSO-*d*_6_): δ 10.34 (s, 1H, NH, exchangeable by D_2_O), 8.70 (t, 1H, *J*^4^ = 2.0 Hz), 8.17 (s, 1H), 8.15 (dt, 1H, *J*^3^ = 8.0 Hz, *J*^4^ = 2.0 Hz), 7.96–7.93 (m, 3H), 7.45–7.42 (m, 3H), 4.31 (br s, 4H), 3.81 (s, 3H), 3.76 (t, 4H, *J*^3^ = 3.6 Hz), 3.54 (s, 2H), 2.37-2.31 (m, 8H), 2.14 (s, 3H) ppm; ^13^C NMR (100 MHz, DMSO-*d*_6_): δ 165.6, 156.7, 153.0, 152.1, 142.23, 141.3, 139.2, 138.8, 133.7, 128.7, 128.4, 127.7, 123.2, 121.9, 119.9, 118.2, 66.3, 61.6, 54.7, 52.6, 45.7, 45.1, 29.5 ppm. MS (ESI) 527 [M^+^]. Anal. calcd for C_29_H_34_N_8_O_2_. 0.6 H_2_O: C, 64.81; H, 6.60; N, 20.85. Found: C, 64.87; H, 6.41; N, 20.65.

##### 4-((4-Methylpiperazin-1-yl)methyl)-*N*-(3-(6-morpholino-9-phenyl-9H-purin-2-yl)phenyl)benzamide (**3b**)

Compound **3b** (0.10 g, 0.16 mmol, 55%) was obtained as an off-white solid from the reaction of **26d** (0.15 g, 0.29 mmol) with *N*-methyl-piperazine (0.89 μL, 0.8 mmol, 5 eq.) in dioxane (0.12 mL) after 50 min. M.p. 219–221 °C. IR (nujol) νmax: 3344, 3098, 2930, 2792, 1651, 1600, 1576, 1531, 1512 cm^−1^. ^1^H NMR (400 MHz, DMSO-*d*_6_): *δ* 10.34 (s, 1H, NH, exchangeable by D_2_O), 8.66 (br. s, 1H), 8.63 (s, 1H), 8.10 (d, 1H, *J*^3^ = 8.0 Hz), 7.98–7.91 (m, 5H), 7.64 (t, 2H, *J*^3^ = 8.0 Hz), 7.51-7.42 (m, 4H), 4.37 (br. s, 4H), 3.80 (t, 4H, *J*^3^ = 4.8 Hz), 3.52 (s, 2H), 2.33 (s, 8H), 2.14 (s, 3H) ppm; ^13^C NMR (100 MHz, DMSO-*d*_6_): *δ* 165.6, 157.4, 153.2, 151.4, 142.2, 139.5, 139.3, 138.6, 135.0, 133.7, 129.6, 128.6, 128.5, 127.7, 127.6, 123.3, 123.2, 122.0, 119.9, 118.8, 66.3, 61.6, 54.7, 52.6, 45.7, 45.3 ppm. MS (ESI) 589 [M^+^]. Anal. calcd for C_34_H_36_N_8_O_2_. 0.1 H_2_O: C, 69.16; H, 6.18; N, 18.98. Found: C, 69.01; H, 6.19; N, 18.90.

##### *N*-(3-(9-Benzyl-6-morpholino-9H-purin-2-yl)phenyl)-4-((4-methylpiperazin-1-yl)methyl)benzamide (**4b**)

Compound **4b** (0.08 g, 0.14 mmol, 48%) was obtained as an off-white solid from the reaction of **26c** (0.16 g, 0.29 mmol) with *N*-methyl-piperazine (78 μL, 0.7 mmol, 5 eq.) in dioxane (1 mL) after 2 h. M.p. 213–215 °C. IR (nujol) νmax: 3284, 2941, 2854, 2797, 2165, 1641, 1604, 1575, 1531 cm^−1^. ^1^H NMR (400 MHz, DMSO-*d*_6_): δ 10.34 (s, 1H, NH, exchangeable by D_2_O), 8.74 (t, 1H, *J*^4^ = 1.6 Hz), 8.33 (s, 1H), 8.14 (dt, 1H, *J*^3^ = 7.6 Hz, *J*^4^ = 2.4 Hz), 7.94 (br.s, 2H), 7.92 (br.s, 1H), 7.46-7.40 (m, 5H), 7.34 (t, 2H, *J*^3^ = 7.2 Hz), 7.27 (t, 1H, *^3^J =* 7.2 Hz), 5.47 (s, 2H), 4.31 (br. s, 4H), 3.76 (t, 4H, *J*^3^ = 4.8 Hz), 3.52 (br. s, 2H), 2.34 (br. s, 8H), 2.14 (s, 3H) ppm; ^13^C NMR (100 MHz, DMSO-*d*_6_): δ 165.6, 156.9, 153.0, 1561.7, 142.2, 140.5, 139.3, 138.7, 137.1, 133.7, 128.7, 128.6, 128.5, 127.9, 127.8, 127.7, 123.2, 121.9, 119.9, 118.2, 66.2, 61.6, 54.7, 52.6, 46.2, 45.7, 45.2 ppm. MS (ESI) 603 [M^+^]. Anal. calcd for C_35_H_38_N_8_O_2_. 1.0 H_2_O: C, 68.54; H, 6.59; N, 17.83. Found: C, 68.32; H, 6.45; N, 18.21.

##### *N*-(3-(9-(3,4-Dimethylphenyl)-6-morpholino-9H-purin-2-yl)phenyl)-4-((4-methylpiperazin-1-yl) methyl)benzamide (**7b**)

Compound **7b** (0.15 g, 0.24 mmol, 77%) was obtained as an off-white solid from the reaction of **26e** (0.17 g, 0.32 mmol) with *N*-methyl-piperazine (176 μL, 1.58 mmol, 5 eq.) in dioxane (2 mL) after 14 h. M.p. 216–218 °C. IR (nujol) νmax: 3281, 1646, 1608, 1575, 1534 cm^−1^. ^1^H NMR (400 MHz, DMSO-*d*_6_) δ: 10.33 (s, 1H, NH, exchangeable by D_2_O), 8.70 (t, 1H, *J*^4^ = 1.6 Hz), 8.56 (s, 1H), 8.09 (dt, 1H, *J*^3^ = 8.0, *J*^4^ = 1.6 Hz), 7.91 (d, 2H, *J*^3^ = 8.0 Hz), 7.90 (m, 1H), 7.74 (d, 1H, *J*^4^ = 2.0 Hz), 7.65 (dd, 1H, *J*^3^ = 8.0, *J*^4^ = 2.0 Hz), 7.5–7.4 (m, 4H), 4.36 (br. s, 4H), 3.80 (t, 4H, *J*^3^ = 4.8 Hz), 3.52 (s, 2H), 2.5–2.3 (m, 8H), 2.36 (s, 3H), 2.31 (s, 3H), 2.14 (s, 3H). ^13^C NMR (100 MHz, DMSO-*d*_6_) δ: 165.58, 157.36, 153.22, 151.42, 142.23, 139.50, 139.27, 138.66, 137.73, 135.92, 133.70, 132.76, 130.33, 128.67, 128.48, 127.68, 124.23, 123.17, 121.99, 120.68, 119.97, 118.73, 66.28, 61.62, 54.70, 52.58, 45.74, 45.24, 19.51, 19.01. MS (ESI) 618 [M^+^ + 1]. HRMS (ESI): *m*/*z* [M^+^ + 1] calcd for C_36_H_41_N_8_O_2_: 617.3352; found: 617.3358.

##### *N*-(3-(9-(3,4-Dimethylphenyl)-6-morpholino-9H-purin-2-yl)phenyl)-4-(morpholinomethyl)benzamide (**7c**)

Compound **7c** (0.13 g, 0.21 mmol, 73%) was obtained as a white solid from the reaction of **26e** (0.15 g, 0.29 mmol) with morpholine (127 μL, 1.46 mmol, 5 eq.) in dioxane (2 mL) after 14 h. M.p. 219–221 °C. IR (nujol) νmax: 3280, 1646, 1607, 1573, 1516 cm^−1^. ^1^H NMR (400 MHz, DMSO-*d*_6_) *δ*: 10.34 (s, 1H, NH, exchangeable by D_2_O), 8.70 (s, 1H), 8.56 (s, 1H), 8.09 (d, 1H, *J*^3^ = 8.0 Hz), 7.92 (d, 2H, *J*^3^ = 8.0 Hz), 7.89 (m, 1H), 7.74 (s, 1H), 7.65 (d, 1H, *J*^3^ = 8.0 Hz), 7.5–7.4 (m, 4H), 4.36 (br. s, 4H), 3.80 (br. s, 4H), 3.6–3.5 (m, 6H), 2.36 (br. s, 7H), 2.31 (s, 3H). ^13^C NMR (100 MHz, DMSO-*d*_6_) *δ*: 165.56, 157.36, 153.23, 151.43, 141.73, 139.51, 139.26, 138.66, 137.74, 135.94, 133.78, 132.76, 130.34, 128.80, 128.50, 127.71, 124.25, 123.19, 122.00, 120.69, 119.98, 118.73, 66.29, 66.20, 61.99, 53.19, 45.30, 19.52, 19.02. MS (ESI) 605 [M^+^ + 1]. HRMS (ESI): *m*/*z* [M^+^ + 1] calcd for C_35_H_38_N_7_O_3_: 604.3036; found: 604.3031.

##### *N*-(3-(9-(3-Fluorophenyl)-6-morpholino-9H-purin-2-yl)phenyl)-4-((4-methylpiperazin-1-yl)methyl)benzamide (**9b**)

Compound **9b** (0.19 g, 0.32 mmol, 84%) was obtained as a light-yellow solid from the reaction of **26f** (0.20 g, 0.38 mmol) with *N*-methyl-piperazine (178 μL, 1.6 mmol, 5 eq.) in dioxane (0.66 mL) after 5 h and 30 min. M.p. 227–229 °C. IR (nujol) νmax: 3370, 3097, 1655, 1609, 1597, 1578, 1534, 1511 cm^−1^. ^1^H NMR (400 MHz, DMSO-*d*_6_): 10.34 (s, 1H, NH, exchangeable by D_2_O), 8.70 (s, 1H), 8.69 (t, 1H, *J*^4^ = 2.0 Hz), 8.10 (dt, 1H, *J*^3^ = 7.6 Hz, *J*^4^ = 1.2 Hz), 7.98-7.91 (m, 5H), 7.69 (dt, 1H, *J*^4^ = 6.4 Hz, *J*^3^ = 8.0 Hz), 7.47 (s, 1H), 7.38 (d, 2H, *J*^3^ = 8.0 Hz), 7.33 (tdd, 1H, *J*^3^ = 8.0 Hz, *J*^4^ = 2.4 Hz, *J*^4^ = 0.8 Hz), 4.36 (br. s, 4H), 3.80 (t, 4H, *J*^3^ = 4.8 Hz), 3.53 (s, 2H), 2.33 (br. s, 8H), 2.16 (s, 3H) ppm; ^13^C NMR (100 MHz, DMSO-*d*_6_): δ 165.6, 162.26 (d, *J^1^ =* 243.0 Hz), 157.6, 153.2, 151.3, 142.2, 139.21, 139.28, 138.5, 136.5 (d, *J*^3^ = 10.0 Hz), 133.7, 131.3 (d, *J*^3^ = 10.0 Hz), 128.7, 128.5, 127.7, 123.1, 122.1, 120.0, 119.0 (d, *J*^4^ = 3.0 Hz), 118.8, 114.4 (d, *J*^2^ = 20.0 Hz), 110.4 (d, *J*^2^ = 26.0 Hz), 66.3, 61.6, 54.6, 52.5, 45.6, 45.3 ppm. MS (ESI) 607 [M^+^]. Anal. calcd for C_34_H_35_FN_8_O_2_. 0.5 H_2_O: C, 66.33; H, 5.89; N, 18.20. Found: C, 66.10; H, 5.66; N, 18.36.

##### *N*-(3-(9-(4-Chlorophenyl)-6-morpholino-9H-purin-2-yl)phenyl)-4-((4-methylpiperazin-1-yl)methyl)benzamide (**10b**)

Compound **10b** (0.18 g, 0.28 mmol, 85%) was obtained as an off-white solid from the reaction of **26g** (0.18 g, 0.33 mmol) with *N*-methyl-piperazine (156 μL, 1.4 mmol, 5 eq.) in dioxane (1 mL) after 6 h. M.p. 244–246 °C. IR (nujol) νmax: 3321, 2949, 2845, 2790, 2760, 1645, 1605, 1570, 1537, 1512 cm^−1^. ^1^H NMR (400 MHz, DMSO-*d*_6_): *δ* 10.34 (s, 1H, NH, exchangeable by D_2_O), 8.64 (br. s, 1H), 8.59 (s, 1H), 8.09 (d, 1H, *J*^3^ = 8.0 Hz), 8.00 (d, 2H, *J*^3^ = 8.8 Hz), 7.94 (d, 1H, *J*^3^ = 8.8 Hz), 7.90 (d, 2H *J*^3^ = 8.4 Hz), 7.67 (d, 2H, *J*^3^ = 8.8 Hz), 7.44–7.40 (m, 3H), 4.33 (br. s, 4H), 3.78 (t, 4H, *J*^3^ = 4.4 Hz), 3.54 (s, 2H), 2.35 (br. s, 8H), 2.13 (s, 3H) ppm; ^13^C NMR (100 MHz, DMSO-*d*_6_): *δ* 165.9, 157.7, 153.3, 151.4, 142.3, 139.4, 139.3, 138.6, 134.02, 133.8, 132.1, 129.7, 128.9, 127.8, 125.1, 123.4, 122.2, 120.1, 118.8, 66.4, 61.7, 54.8, 52.6, 45.8, 45.4 ppm. MS (ESI) 623 [M^+^]. Anal. calcd. for C_34_H_35_ClN_8_O_2_. 1.0 H_2_O: C, 63.69; H, 5.82; N, 17.48. Found: C, 63.64; H, 5.53; N, 17.4.

##### *N*-(3-(9-(4-Benzamidophenyl)-6-morpholino-9H-purin-2-yl) phenyl)-4-((4-methylpiperazin-1-yl)methyl)benzamide (**14b**)

Compound **14b** (0.05 g, 0.07 mmol, 36%), was obtained as an off-white solid from the reaction of **26h** (0.13 g, 0.20 mmol) with *N*-methyl-piperazine (109 μL, 0.98 mmol, 5 eq.) in dioxane (1 mL) after 31 h. M.p. 273–275 °C. IR (nujol) νmax: 3333, 1675, 1640, 1584, 1534, 1508 cm^−1^. ^1^H NMR (400 MHz, DMSO-*d*_6_) *δ*: 10.48 (s, 1H, NH, exchangeable by D_2_O), 10.35 (s, 1H, NH, exchangeable by D_2_O), 8.65 (t, 1H, *J*^4^ = 2.0 Hz), 8.60 (s, 1H), 8.12 (dt, 1H, *J*^3^ = 8.0, *J*^4^ = 1.2 Hz), 8.05–7.91 (m, 9H), 7.62 (dt, 1H, *J*^3^ = 8.4 Hz, *J*^4^ = 1.6 Hz), 7.55 (t, 2H, *J*^3^ = 8.4 Hz), 7.44 (t, 1H, *J*^3^ = 8.0 Hz), 7.43 (d, 2H, *J*^3^ = 8.4 Hz), 4.37 (br. s, 4H), 3.80 (t, 4H, *J*^3^ = 4.8 Hz), 3.51 (s, 2H), 2.32 (br. s, 8H), 2.13 (s, 3H). ^13^C NMR (100 MHz, DMSO-*d*_6_) *δ*: 165.76, 165.66, 157.43, 153.23, 151.44, 142.21, 139.47, 139.27, 138.65, 138.57, 134.73, 133.73, 131.76, 130.44, 128.66, 128.50, 128.46, 127.73, 127.70, 123.77, 123.28, 122.04, 121.07, 119.95, 118.69, 66.29, 61.62, 54.70, 52.57, 45.74, 45.18. MS (ESI) 708 [M^+^ + 1]. HRMS (ESI): *m*/*z* [M^+^ + 1] calcd for C_41_H_42_N_9_O_3_: 708.3411; found: 708.3416. 

#### 2.2.7. Synthesis of (4-((3-(9-(3,4-Dimethylphenyl)-6-morpholino-9H-purin-2-yl)phenyl)carbamoyl)phenyl)methanaminium Chloride (**7a**)

Hydrazine monohydrate (40 eq., 370 µL, 7.62 mmol) was added to a suspension of **27** (0.13 g, 0.19 mmol) in dioxane (1.5 mL). The reaction was carried out in a closed vial at 60 °C under efficient magnetic stirring and was carefully monitored by TLC. After 2.5 h, the absence of the starting reagent **27** was confirmed. The solution was cooled down to room temperature, and 45 mL of HCl (1M) was added to the solution under efficient magnetic stirring. A white solid started to precipitate gradually from the solution. After two days, the solid in suspension was filtered off and recrystallised in acetone. The resulting white solid was identified as **7a** (68 mg, 0.12 mmol, 63%). M.p. 256–258 °C. IR (nujol) νmax: 2851, 2622, 1677, 1610, 1592, 1550, 1507 cm^−1^. ^1^H NMR (400 MHz, DMSO-*d*_6_) *δ*: 10.47 (s, 1H, NH, exchangeable by D_2_O), 8.74 (t, 1H, *J*^4^ = 2.0 Hz), 8.60 (br. s, 3H, NH_3_^+^, exchangeable by D_2_O), 8.57 (s, 1H), 8.10 (dt, 1H, *J*^3^ = 8.0 Hz, *J*^4^ = 1.2 Hz), 8.03 (d, 2H, *J*^3^ = 8.4 Hz), 7.94 (ddd, 1H, *J*^3^ = 8.0 Hz, *J*^4^ = 2.0 Hz, *J*^4^ = 1.2 Hz), 7.76 (d, 1H, *J*^4^ = 2.0 Hz), 7.66 (m, 3H), 7.44 (t, 1H, *J*^3^ = 8.0 Hz), 7.38 (d, 1H, *J*^3^ = 8.4 Hz,), 4.36 (br. s, 4H), 4.11 (q, 2H, *J*^3^ = 6.0 Hz), 3.80 (t, 4H, *J*^3^ = 4.8 Hz), 2.36 (s, 3H), 2.31 (s, 3H). ^13^C NMR (100 MHz, DMSO-*d*_6_) *δ*: 165.07, 157.29, 153.18, 151.37, 139.47, 139.13, 138.62, 137.68, 137.50, 135.86, 134.76, 132.73, 130.29, 128.76, 128.45, 127.92, 124.18, 123.26, 122.01, 120.62, 120.00, 118.68, 66.25, 45.24, 41.75, 19.47, 18.98. MS (ESI) 535 [M^+^ + 1]. Anal. Calcd for C_31_H_31_ClN_7_O_2_.HCl: C, 65.31; H, 5.66; N, 17.20; found: C, 65.22; H, 5.62; N, 17.35.

#### 2.2.8. Synthesis of 4-(Benzamidomethyl)-*N*-(3-(9-(3,4-dimethylphenyl)-6-morpholino-9H-purin-2-yl)phenyl)benzamide (**7e**)

A mixture of **7a** (26.8 mg, 0.05 mmol) and Et_3_N (5 eq., 33 µL, 0.24 mmol) was suspended in dry dioxane (3 mL), and benzoic anhydride (2 eq., 21.3 mg, 0.09 mmol) was added. The reaction was carried out in a closed vial at 80 °C under efficient magnetic stirring and was carefully monitored by TLC. After 2 h, the absence of the starting reagent **7a** was confirmed. The reaction was cooled down to room temperature. Then, distilled water and ethanol were added to the solution until a white solid precipitated. The resulting suspension was cooled down in an ice bath, and the solid was filtered off and washed successively with water, cold ethanol, and diethyl ether. The resulting off-white solid was identified as **7e** (21.0 mg, 0.03 mmol, 70%). M.p. 183–185 °C. IR (nujol) νmax: 3289, 2858, 1645, 1607, 1573, 1539, 1515 cm^−1^. ^1^H NMR (400 MHz, DMSO-*d*_6_) *δ*: 10.33 (s, 1H, NH, exchangeable by D_2_O), 9.13 (t, 1H, *J*^3^ = 6.0 Hz, NH, exchangeable by D_2_O), 8.70 (t, 1H, *J*^4^ = 1.6 Hz), 8.56 (s, 1H), 8.09 (dt, 1H, *J*^3^ = 8.0 Hz, *J*^4^ = 1.6 Hz), 7.95 (m, 5H), 7.75 (d, 1H, *J*^4^ = 2.0 Hz), 7.65 (dd, 1H, *J*^3^ = 8.4 Hz, *J*^4^ = 2.0 Hz), 7.55 (dt, 1H, *J*^3^ = 7.6 Hz, *J*^4^ = 1.2 Hz), 7.50 (m, 4H), 7.43 (t, 1H, *J*^3^ = 8.0 Hz), 7.37 (d, 1H, *J*^3^ = 8.4 Hz), 4.56 (d, 2H, *J*^3^ = 6.0 Hz), 4.35 (br. s, 4H), 3.79 (t, 4H, *J*^3^ = 4.8 Hz), 2.36 (s, 3H), 2.30 (s, 3H). ^13^C NMR (100 MHz, DMSO-*d*_6_) *δ*: 166.32, 165.47, 157.32, 153.20, 151.40, 143.45, 139.47, 139.22, 138.63, 137.70, 135.89, 134.23, 133.49, 132.74, 131.32, 130.30, 128.46, 128.36, 127.79, 127.24, 127.04, 124.21, 123.16, 121.98, 120.64, 119.96, 118.71, 66.26, 45.22, 42.44, 19.48, 18.98. MS (ESI) 638 [M^+^ + 1]. HRMS (ESI): *m*/*z* [M^+^ + 1] calcd for C_38_H_36_N_7_O_3_: 638.2880; found: 638.2883.

## 3. Results and Discussion

### 3.1. Rational Design and Modelling

**Figure 4:**
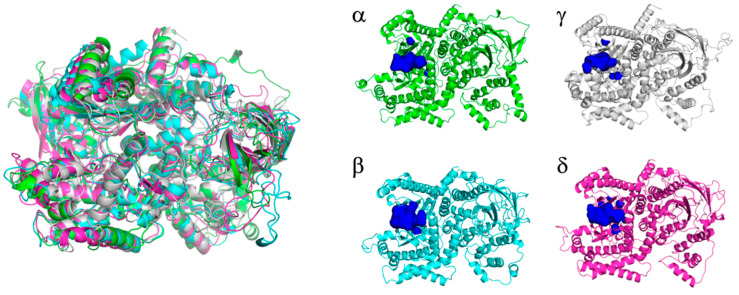
On the left, the structural alignment of the tertiary structures of PI3Kα (green), PI3Kβ (cyan), PI3Kγ (grey), and PI3Kδ (magenta). On the right, activity pockets are highlighted as blue surfaces for each isoform.

An alignment of the tertiary structures was performed, confirming the active site’s conservation in all four PI3K isoforms. Figure 4 shows this alignment, revealing an optimal overlap with enzymes in different colours, where only the most mobile parts (loops and bends) are distorted. These 3D structures were predicted by using the SWISS-MODEL homology-modelling server to fulfil the existing gaps while maintaining high structural alignment to their respective PDB structures 6PYS (a), 4BFR (b), 6AUD (g), and 6TNR (d). Images on the right allow one to observe the activity pocket availability and the overall conservation among isoforms. Nevertheless, receptors α and γ are similar to each other, presenting a smaller pocket, whilst pockets of isoforms β and δ are slightly larger. The size/volume of the binding pocket can be very important for the activity, favouring or not favouring a tight binding from which the ligand does not leave easily. 

A virtual screening of some of the synthetic precursors **25** studied in this work was performed on the four targets under study due to the optimal structural similarity with the already reported pharmacophore (Table S2). Analysis of the binding pattern of these synthetic precursors was carried out (Figure S1), and the results were in accordance with the reported interactions for PI3Kα [3,22]. Hence, in an attempt to increase the affinity of the ligands while considering this scaffold, a ligand-based and target-based rational drug design strategy was developed simultaneously in the design of the library of ligands presented in Table 1. The incorporation of bulky/small, polar/apolar groups such as R^1^ and R^2^ was considered in combination with the aimed interactions. Table 1 also shows, for each ligand, a diverse range of descriptions of their respective physicochemical and structural properties, as well as the ΔG_binding_ score for each isoform gathered, aiming at the identification of potentially selective ligands, and evaluating structural similarities that may indicate an interaction pattern that justifies the selectivity obtained.

In this library of 105 ligands, the same scaffold was maintained, and variations in the substituent groups (R^1^ and R^2^) are shown in Table 1. Here, the criterion defined for a ligand to be considered selective was that the difference between its ∆G_binding_ in one isoform and in the remaining targets must be equal to or higher than 1 kcal/mol [∆ (∆G_binding_) ≥ 1 Kcal/mol], as the molecules are not very flexible (few rotatable bonds); thus, the affinity error estimation is minimised [29,48]. The docking calculations were all conducted on the same computer, with no variation in processor/performance. 

**Figure 5:**
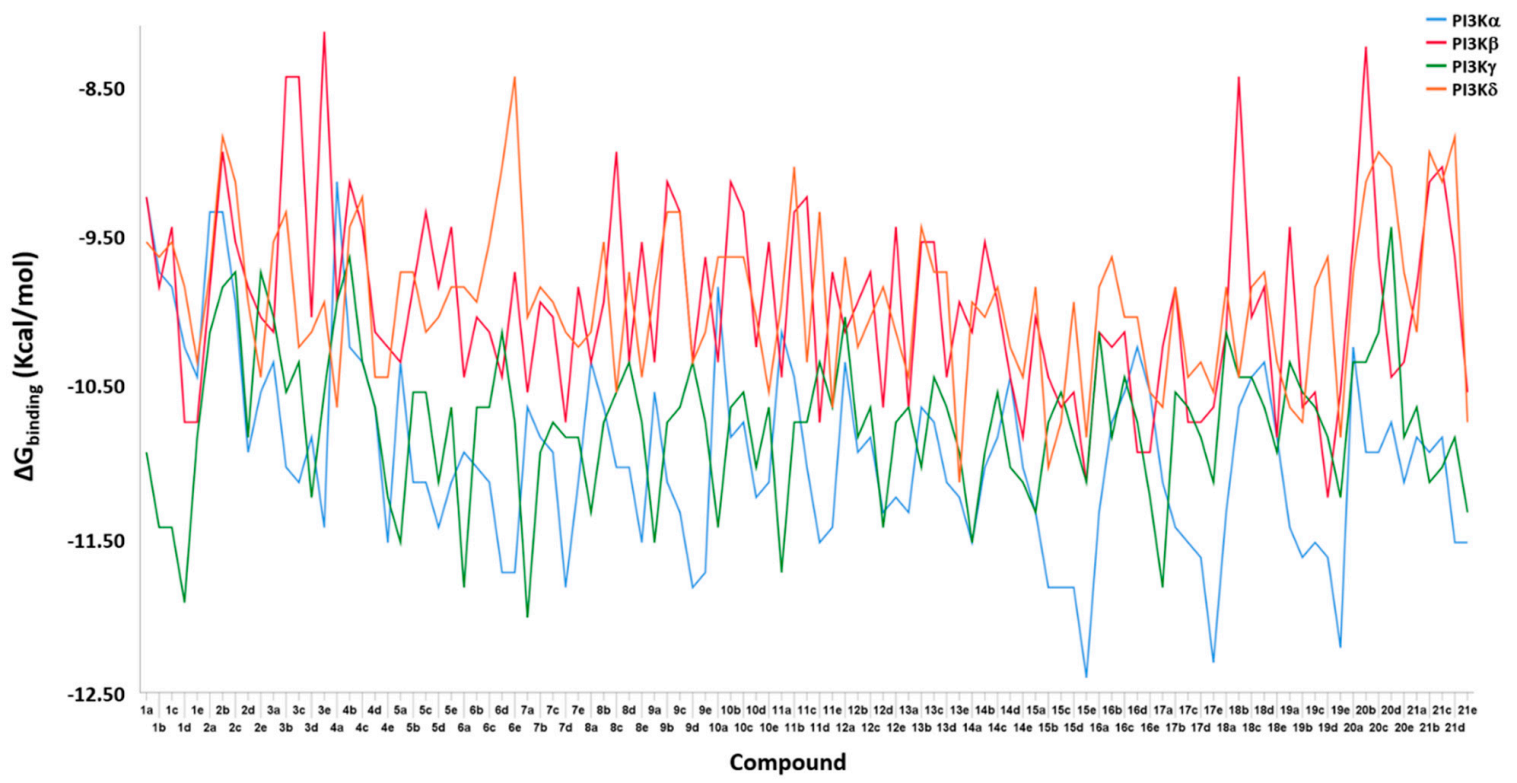
Graphical representation of the binding energies for the designed library of ligands in the 4 isoforms of PI3K. Blue line—PI3Kα; red line—PI3Kβ; green line—PI3Kγ; orange line—PI3Kδ.

The graphic in Figure 5 depicts the ∆G_binding_ profile of the ligands for each of the four targets under study. It shows an overall higher affinity of ligands for isoform PI3Kα, followed by PI3Kγ isoform, represented by the blue and green lines, respectively. These results agree with the information reported in the literature for ligands with this type of scaffold [3,22], which indicates that ligands exhibit preferential selectivity for the α and γ isoforms of PI3K. By following the established criteria for selectivity, the VS yielded 19 selective ligands, representing 18% of the library. 

**Figure 6:**
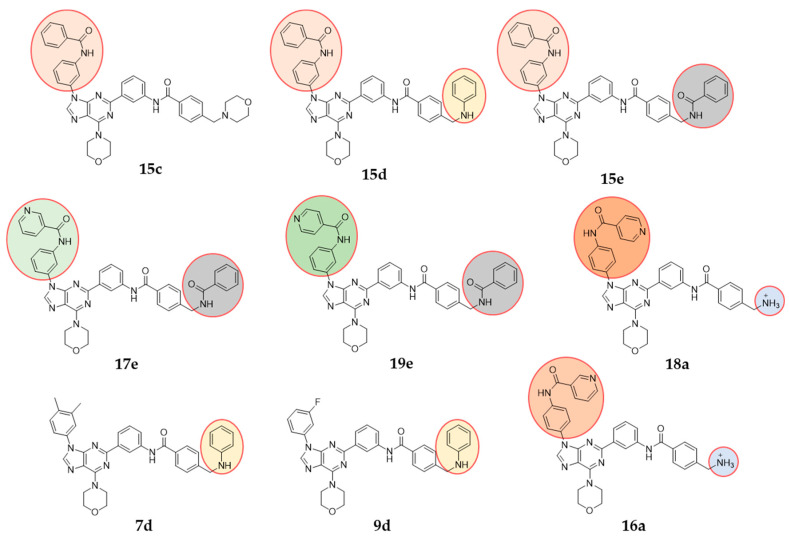
Set of selective ligands found for PI3Kα.

**Figure 7:**
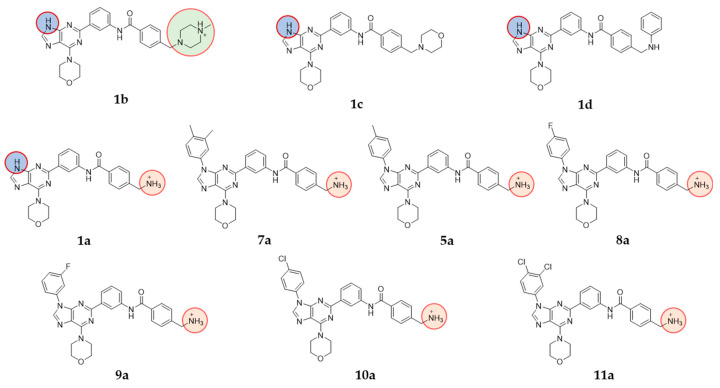
Set of selective ligands found for PI3Kγ.

From these, 9 ligands were found to be selective for PI3Kα (**7d**, **9d**, **15c**, **15d**, **15e**, **16a**, **17e**, **18a**, and **19e**) and 10 for PI3Kγ (**1a**, **1b**, **1c**, **1d**, **5a**, **7a**, **8a**, **9a**, **10a**, and **11a**), whose structures are presented in Figure 6 and Figure 7, respectively. The most promising selective ligands are **9d**, **1b**, and **1c**, which present a ∆ (∆G_binding_) equal or greater than 1.5 kcal/mol.

Analysing the chemical structure of the PI3Kα selective ligands, a clear pattern is noticeable for the R^1^ group (pink, orange, and green circles) since 78% of these ligands have an amide functional group connecting two aromatic rings. When the amide function is in the *meta* position (**15c**, **15d**, **15e**, **17e**, **19e**), the R^2^ group (grey circle) is mostly highly voluminous substituents (NHPh and NHCOPh) and nonprotonated at physiological pH. When the same functional group is in the *para* position (**18a**, **16a**), the R^2^ group (light-blue circle) is the protonated primary amine (^+^NH_3_). As for ligands **7d** and **9d**, an aniline (cream-coloured circle) in R^2^ is preferable when simpler or less voluminous groups are R^1^. 

The chemical structure analysis of PI3Kγ selective ligands show the pattern of the R^2^ group (pink circles). Except for ligands **1c** and **1d**, all present a protonated R^2^ group at physiological pH. Another consistency in the results for this isoform of PI3K is the selectivity shown by ligands whose R^1^ group (blue circles) is a hydrogen atom. In contrast with PI3Kα selective ligands, the PI3Kγ selective ligands present a less voluminous R^1^ group (pink and green circles versus blue circles). 

**Figure 8:**
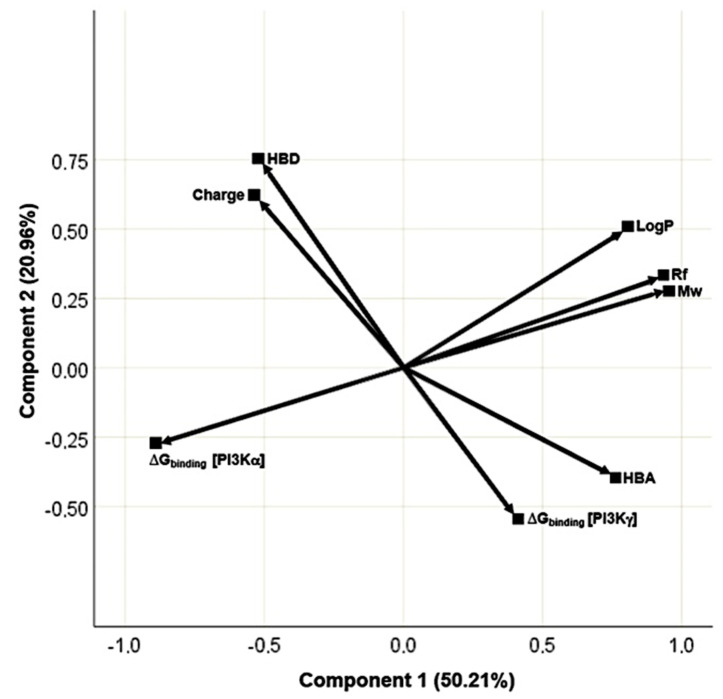
Principal component analysis (PCA) biplot of the first two components, objects factor scores, and loadings of data presented in Table 1. Rotated component matrix and scores were calculated using IBM SPSS Statistics software.

To ascertain the correlation between all the ligands’ physicochemical and structural properties and the ∆G_binding_ for the α and γ isoforms of PI3K (Table 1), a principal component analysis (PCA) of the data was performed and is presented in Figure 8. 

Here, PC1 represents 50.21% and PC2 20.96%. Thus, the first two components describe over 71% of data. Figure 8 shows that the properties that most influence the affinity of the ligands toward the PI3Kα are LogP, Rf, and MW, as large angles (approaching 180°) are observed between the vectors representing these properties and the one for ∆G_binding_ of PI3Kα. This suggests that more hydrophobic and voluminous ligands present better affinity toward the α isoform. In contrast, for PI3Kγ, the ligands’ affinity is more influenced by the charge of the ligand at physiological pH and the number of HBD. In fact, 75% of the selective ligands found for PI3Kγ are protonated at physiological pH.

**Figure 9:**
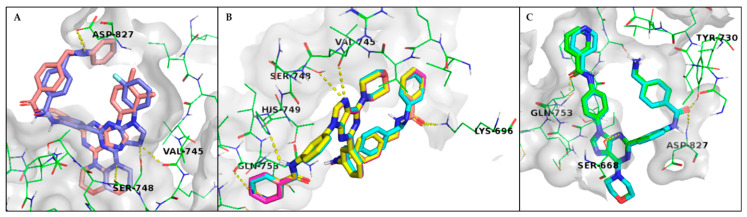
Interaction-binding mode of docked ligands in the active site of PI3Kα. Overlay of ligands **7d** and **9d** (**A**); ligands **15d**, **15e**, **17e**, and **19e** (**B**); ligands **16a** and **18a** (**C**).

**Figure 10:**
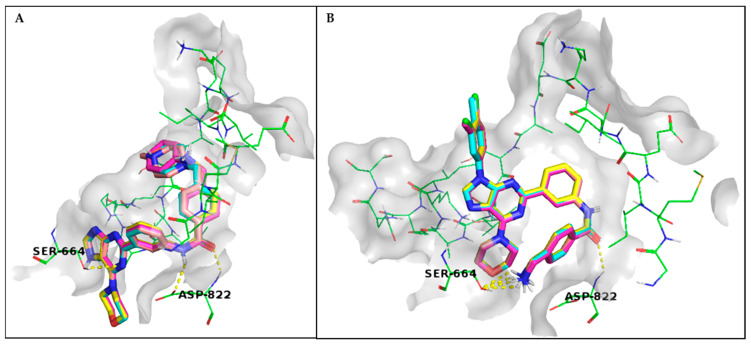
Interaction-binding mode of docked ligands in the active site of PI3Kγ. Overlay of the selective ligands **1a**, **1b**, **1c**, and **1d** (**A**); ligands **5a**, **7a**, **8a**, **9a**, **10a**, and **11a** (**B**).

The binding pattern for the selective ligands was studied for the α and γ isoforms of PI3K aiming to identify key interactions reported with selective inhibitors [21]. Figure 1 highlights the differences among the binding pockets’ sequence of each isoform, which helps to perceive the differences in amino acids assignments in Figure 9 and Figure 10.

The interaction of the selective ligands found for PI3Kα in the target’s active site is depicted in Figure 9. Ligands **7d** and **9d** have the R^2^ group overlapped, establishing a common interaction at the active site of PI3Kα, while the remaining part of the molecules are slightly shifted (Figure 9A). Except for ligand **15c**, the set of ligands in which the R^1^ group is *meta*-substituted (**15d**, **15e**, **17e**, and **19e**) overlap very well (Figure 9B). The same is depicted in Figure 9C for the *para*-substituted R^1^ ligands with the primary protonated amine in R^2^ (**16a** and **18a**). Each ligand, **9d**, **15e**, and **16a**, was used as a model to study the interaction pattern of these ligands in the active site of PI3Kα.

Ligand **9d** is an acceptor to three hydrogen bonds from SER-742 and VAL-745 and an HBD to ASP-827 (Figure 9A). In contrast, the polar interactions established by **15e** in the active site of PI3Kα (Figure 9B) do not match any of the reported key amino acids to confer selectivity. However, for ligand **19e**, a key interaction with GLN-753 is observed with the nitrogen on the pyridine ring in the R^1^ substituent group. The same happens for ligand **16a** and **18a**, since they both establish a key hydrogen bond with GLN-753 in the active site of PI3Kα (Figure 9C). Here, the carbonyl group from the R^1^ substituent acts as an HBA in this interaction.

Regarding PI3Kγ, it is possible to verify in Figure 10A an optimal overlap of the ligands containing a hydrogen atom in the R^1^ substituent group (**1a**, **1b**, **1c**, and **1d**). On the other hand, ligands with an aromatic group in R^1^ and the protonated primary amine in R^2^ (**5a**, **7a**, **8a**, **9a**, **10a**, and **11a**) also show an optimal overlap in Figure 10B. 

The interaction-binding mode of ligands **1b** and **1c** (Figure 10A) and ligand **7a** (Figure 10B) in the active site of PI3Kγ were also studied. In both interactions, the carbonyl group acts as an HBA for ASP-822. Another common interaction is established toward SER-664. However, different parts of the ligands interact with it. While ligands **1b** and **1c** act as HBA in N-1 of the purine ring, ligand **7a** acts as a HBD to the same residue in the protonated primary amine in R^2^.

It is known that the cancer cells tend to acidify the medium, and this may lead to ligands’ interactions alteration [49]. The analysis of the ligands’ interactions with the PI3Kα (Figure 9) reveals an HBD interaction between the amino group of R^2^ with Asp429 residue and two HBD interactions between the N7 nitrogen with Val745 and Ser748. If medium acidification takes place, amino protonation is expected, and increased interaction of ionic character may occur. The interactions with PI3Kγ (Figure 10) occur at Asp822 (HBA) and Ser664 (HBD). In this case, acidification will not alter the interactions, as the amide function will not be protonated and the terminal amine is already protonated.

### 3.2. Synthesis 

**Scheme 1:**
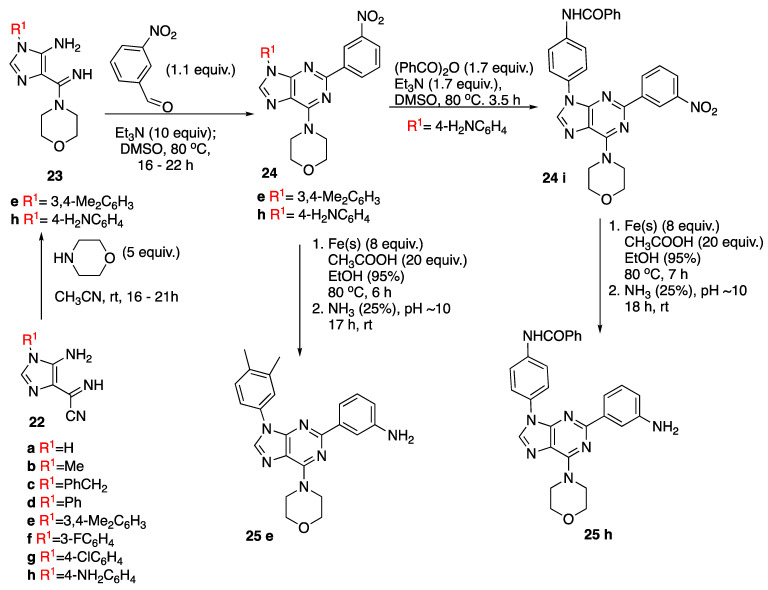
Synthesis description of compounds **22**, **23**, **24** and **25**.

**Scheme 2:**
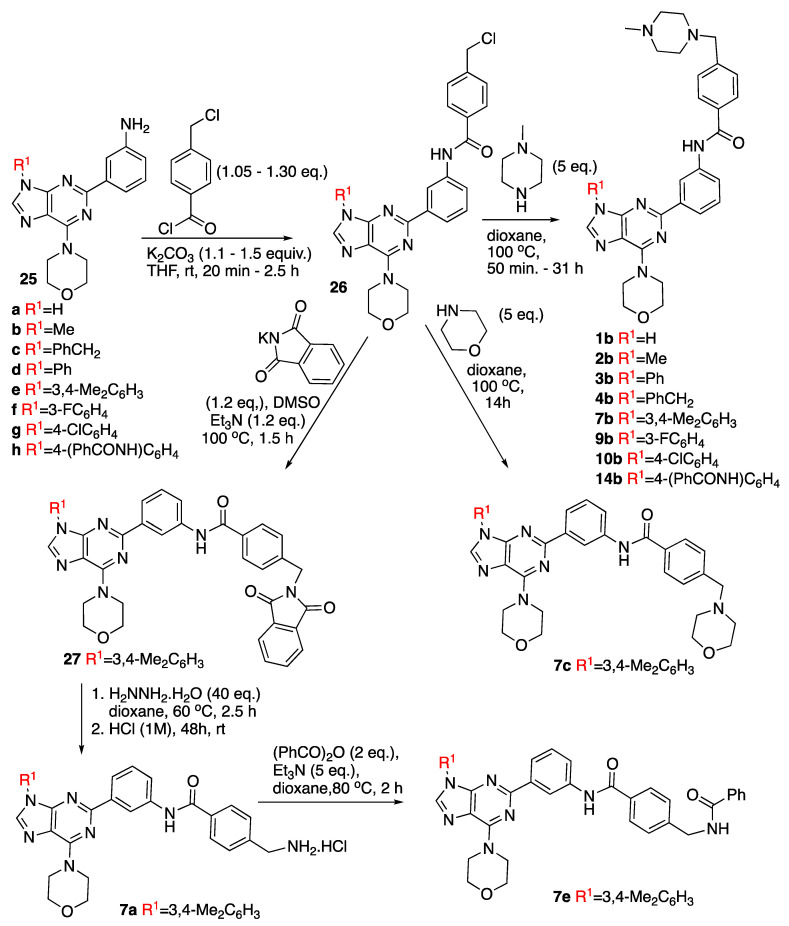
Synthesis description of compounds **26** and the PI3K inhibitors.

After the rational design of selective ligands, the next step was establishing the synthetic pathway to generate the target molecules which have a common scaffold with two substituent groups R^1^ and R^2^ (Figure 3). The selective class I PI3K inhibitors designed were synthesised following the approach presented in Scheme 1 and Scheme 2.

A set of derivatives with different R^1^ (H, alkyl, Aryl) and R^2^ (H, 4-methyl piperazinyl, morpholinyl and amide) were selected to establish the general path to synthesise the compounds. 

Compounds of structure **22a**–**g**, **23a**–**d,f,g**, and **24a**–**d,f,g** were previously described and synthesised according to reported procedures [43,45,46,47]. The new derivatives **23e,h**, **24e,h,i**, and **25e,h** were synthesised using the same experimental reaction conditions (Scheme 1). 

Compounds **22** in the presence of excess morpholine at room temperature generated compounds **23** in high yields (Table S3). Compounds **23** reacted with 3-nitrobenzaldehyde in a basic medium at 80 °C to generate compounds **24e,h**. The reactions take between 16–22 h, and the products were collected after adding water to the reaction mixture. Compound **24i** was achieved by reacting compound **24h** with benzoic anhydride at 80 °C for 3.5 h in the presence of triethylamine.

The method for nitro group reduction of adenine derivatives **24** was described previously and involves the reaction with iron powder in 70% aqueous ethanol and acidic medium under reflux [47]. The reaction workup described was somewhat tedious, as it involved filtrations and extractions, and in this work, we developed a simpler isolation protocol as described hereafter. To synthesise compounds **25**, we followed the protocol described previously, but we used ethanol 95% as the solvent instead of ethanol 70%, and when TLC indicated the absence of the limiting reagent, we added aqueous ammonia solution (25%) to the reaction mixture to raise pH to ~10–11, and it was kept at room temperature in a flask for 12–18 h. During this period, all iron complexes precipitated out of solution and could be eliminated by simple filtration through a diatomaceous earth column.

Elimination of ethanol followed by water addition to the residue allowed us to obtain pure compounds **25** by filtration in excellent yield. The reaction of compounds **25a**–**h** with 4-(chloromethyl)benzoyl chloride for 20 min. to 2.5 h in the presence of potassium carbonate at room temperature generated compounds **26a**–**h** in good to excellent yields. Compounds **1b**–**4b**, **7b,c**, **9b**, **10b**, and **14b** were synthesised by reacting compounds **26a**–**h** with an excess of a secondary amine (4-methyl piperazine or morpholine) in dioxane at 100 ^o^C followed by the addition of water. The products precipitated from the solution and were isolated pure by filtration. To obtain compounds **7a,e**, precursor **27** was initially synthesised. It was achieved from the reaction of **26e** with potassium phthalimide, using triethyl amine as the base and DMSO as the solvent at 100 °C. Reacting compound **27** with a large excess of hydrazine hydrate in dioxane at 60 °C, followed by a reaction with aqueous hydrochloric acid at room temperature, compound **7a** was isolated as the hydrochloric salt in good yield. Compound **7a** was then treated with benzoic anhydride in dioxane at 80 °C in the presence of triethylamine, and compound **7e** was isolated by filtration after precipitation from the reaction mixture by adding water and ethanol. 

### 3.3. Characterisation of PI3K Inhibitors

All the new synthesised compounds were characterised by the usual characterisation methodologies. In particular, synthesised selective class I PI3K inhibitors were fully characterised by physical, analytical, and spectroscopic techniques, including two-dimensional (2D) NMR HSQC and HMBC techniques. The purity of the compounds was evaluated by melting points (M.p.) together with elemental analysis or high-resolution mass spectrometry (HRMS). The pure small molecules usually melted in the range of 2 °C. The analytic data for each compound were accepted when the obtained carbon, hydrogen, and nitrogen values were within 0.4% of the calculated values for the proposed formulas. To support the structure, ^1^H and ^13^C NMR spectra analyses were fundamental. The compounds with the same base structure present similar ^1^H and ^13^C NMR data. 

**Figure 11:**
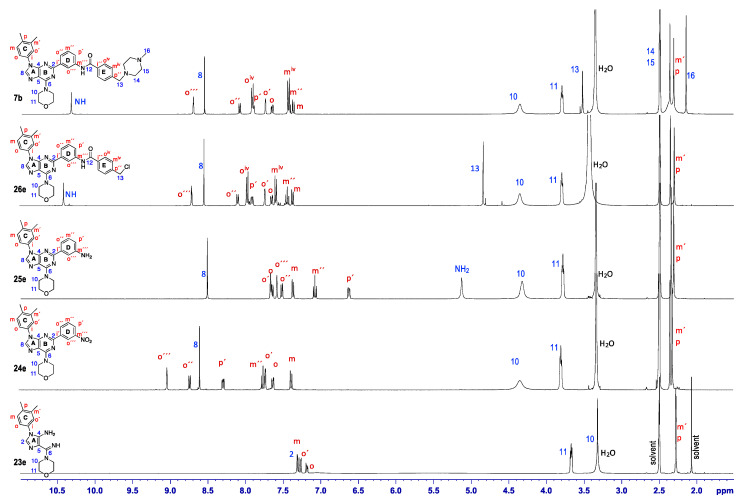
Spectra of ^1^H NMR (DMSO-*d*_6_, 400 MHz) of model compounds with the assignment of signals to the corresponding protons in each structure.

Figure 11 presents the set of ^1^H NMR spectra, from starting reagent **23e** to the final inhibitor **7b**, showing the major changes in ^1^H NMR spectra that occur with increased structural complexity. Figure 11 will be used as a model to explain the assignment of ^1^H NMR signals to the protons in the structures. Compound **23e** presents rings A and C. The ^1^H NMR spectrum of **23e** shows the signal of H2 of ring A at *δ* ~ 7.3 ppm. The signals of ring C protons appear between 7.3 ppm > *δ* > 7.1 ppm with the expected pattern, and at *δ* ~ 2.2 ppm, the signals of the methyl groups appear (Me-p and Me-m′). The signals at *δ* ~ 3.7 ppm and *δ* ~ 3.3 ppm indicate the presence of morpholine moiety, respectively, H10 and H11. Compound **24e** has additional rings B and D, and the system’s aromaticity increased a lot, as revealed by the shift of the signals of compound **24e** to the lower field. The effect of ring current of rings A and B over protons o″ and o‴ together with the presence of a nitro group as a substituent in ring D led to the deprotection of protons of this ring that appear at *δ* ~ 9.1, 8.7, 8.5, and 7.8 ppm. As expected, proton H10 also shifted to a lower field due to the A + B ring current. In the spectrum of compound **25e**, the shift of signals of protons o″, m″, p′, and o‴, together with the appearance of the new signal at *δ* ~ 5.1 ppm that disappears after the addition of deuterated water, confirms the presence of the amino group in ring D. Compound **26e** presents an extra ring E, and the amino group acylated. The presence of new signals at *δ* ~ 10.4, 7.9, 7.6, and 4.8 ppm, assigned to NH, Ho^iv^, Hm^iv^, and H13, support the structure. Compound **7b** has a methyl piperazine unit that was added to system **26e**. Its presence is evidenced by the appearance of the signals at *δ* ~ 2.1 and 2.5 ppm, H16, and H14 + H15, respectively, and the signal of H13 that shifted to *δ* ~ 3.5 ppm. The signal at *δ* ~ 2.5 ppm, coincident with the solvent pick, was confirmed by analysis of the two-dimensional spectra HSQC.

## 4. Conclusions

In summary, a set of new 2,9-disubstituted-6-morpholino purine derivatives was designed by virtual screening and modelling of class I PI3K isoforms. The new inhibitors present high ΔG_binding_ affinity to the class I isoforms of PI3K. The affinity depends on the groups present at N9 and at C2 in the purine main scaffold. The presence of an alkyl or aryl subunit at N9 and the 4-(R^2^-methyl)benzamide generally led to compounds with higher affinity than the parent scaffold (compounds **25**, Scheme 2). PCA analysis and the visual inspection of interactions allowed us to conclude that there are physicochemical and structural properties responsible for selectivity. Consequently, for PI3Kα, the properties that most influence the affinity of the ligands are LogP, Rf, and MW, i.e., affinity increase with bulky and hydrophobic groups. However, for PI3Kγ, the ligands’ affinity is more influenced by the charge and the number of HBD, as the selective ligands are protonated at physiological pH. From the 105 designed compounds, 19 showed selectivity for a single isoform: nine for isoform α (8.6%) and ten for isoform γ (9.5%), and three ligands were identified as the most promising as present Δ(ΔG_binding_) equal to or greater than 1.5 Kcal/mol.

The synthetic pathway to obtain the inhibitors was also proposed, and a set of derivatives were synthesised following that approach. The compounds were obtained in good to excellent yields using simple and efficient isolation techniques. 

This work is a first step in understanding the relationship between the physicochemical properties of the ligands under study and their interaction with the different class I PI3K isoforms, which can aid in the further development of isoform-specific PI3K inhibitors. Nevertheless, future steps in this research will go through the biological evaluation of the ligands hereby described. Then, a more complex study will take place to access an optimal correlation between the docking score and the biological activity of these ligands by applying molecular dynamics techniques and ADMET studies.

## Figures and Tables

**Table 1:** Description of the physicochemical and structural properties of the ligands under study and their affinity (ΔG_binding_) to the 4 Class 1 PI3K isoforms. Selective inhibitors are highlighted in the grey scale.

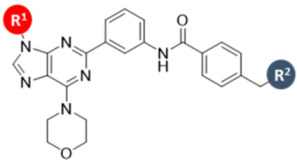							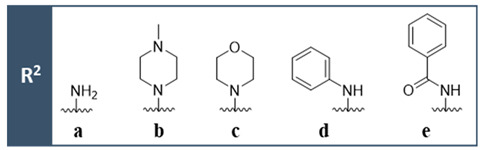				
R^1^	R^2^	Physicochemical and Structural Properties						ΔG_binding_ (kcal/mol)			
		Charge	HBA	HBD	LogP	Mw	Rf	PI3Kα	PI3Kβ	PI3Kγ	PI3Kδ
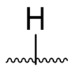 **1**	**a**	+1	8	5	2.33	429.48	134.82	−9.2	−9.2	−10.9	−9.5
	**b**	+1	9	3	3.40	512.62	160.94	−9.7	−9.8	−11.4	−9.6
	**c**	0	11	2	3.28	499.58	153.96	−9.8	−9.4	−11.4	−9.5
	**d**	0	9	3	4.93	505.58	161.15	−10.2	−10.7	−11.9	−9.8
	**e**	0	10	3	4.38	533.59	164.93	−10.4	−10.7	−10.8	−10.3
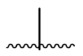 **2**	**a**	+1	8	4	3.01	443.51	139.72	−9.3	−9.8	−10.1	−9.7
	**b**	+1	9	2	3.64	526.65	165.84	−9.3	−8.9	−9.8	−8.8
	**c**	0	11	1	3.52	513.60	158.86	−9.9	−9.5	−9.7	−9.1
	**d**	0	9	2	5.16	519.61	166.05	−10.9	−9.8	−10.8	−9.9
	**e**	0	10	2	4.62	547.62	169.83	−10.5	−10.0	−9.7	−10.4
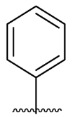 **3**	**a**	+1	8	4	4.66	505.58	169.81	−10.3	−10.1	−10.0	−9.5
	**b**	+1	9	2	5.29	588.72	195.93	−11.0	−8.4	−10.5	−9.3
	**c**	0	11	1	5.17	575.67	188.96	−11.1	−8.4	−10.3	−10.2
	**d**	0	9	2	6.81	581.68	196.15	−10.8	−10.0	−11.2	−10.1
	**e**	0	10	2	6.27	609.69	199.93	−11.4	−8.1	−10.5	−9.9
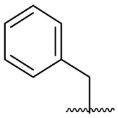 **4**	**a**	+1	8	4	4.74	519.61	164.33	−9.1	−9.9	−9.9	−10.6
	**b**	+1	9	2	5.37	602.74	190.45	−10.2	−9.1	−9.6	−9.4
	**c**	0	11	1	5.25	589.70	183.47	−10.3	−9.4	−10.3	−9.2
	**d**	0	9	2	6.89	595.71	190.66	−10.6	−10.1	−10.6	−10.4
	**e**	0	10	2	6.35	623.72	194.44	−11.5	−10.2	−11.2	−10.4
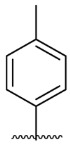 **5**	**a**	+1	8	4	5.16	519.61	174.85	−10.3	−10.3	−11.5	−9.7
	**b**	+1	9	2	5.79	602.74	200.97	−11.1	−9.8	−10.5	−9.7
	**c**	0	11	1	5.67	589.70	194.00	−11.1	−9.3	−10.5	−10.1
	**d**	0	9	2	7.31	595.71	201.19	−11.4	−9.8	−11.1	−10.0
	**e**	0	10	2	6.77	623.72	204.97	−11.1	−9.4	−10.6	−9.8
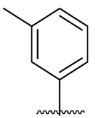 **6**	**a**	+1	8	4	5.16	519.61	174.85	−10.9	−10.4	−11.8	−9.8
	**b**	+1	9	2	5.79	602.74	200.97	−11.0	−10.0	−10.6	−9.9
	**c**	0	11	1	5.67	589.70	194.00	−11.1	−10.1	−10.6	−9.5
	**d**	0	9	2	7.31	595.71	201.19	−11.7	−10.4	−10.1	−9.0
	**e**	0	10	2	6.77	623.72	204.97	−11.7	−9.7	−10.7	−8.4
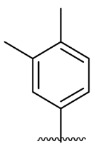 **7**	**a**	+1	8	4	5.66	533.64	179.90	−10.6	−10.5	−12.0	−10.0
	**b**	+1	9	2	6.29	616.77	206.02	−10.8	−9.9	−10.9	−9.8
	**c**	0	11	1	6.17	603.73	199.04	−10.9	−10.0	−10.7	−9.9
	**d**	0	9	2	7.81	609.73	206.23	−11.8	−10.7	−10.8	−10.1
	**e**	0	10	2	7.27	637.74	210.01	−11.1	−9.8	−10.8	−10.2
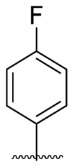 **8**	**a**	+1	8	4	4.81	523.57	170.03	−10.3	−10.3	−11.3	−10.1
	**b**	+1	9	2	5.44	606.71	196.15	−10.6	−9.9	−10.7	−9.5
	**c**	0	11	1	5.32	593.66	189.17	−11.0	−8.9	−10.5	−10.5
	**d**	0	9	2	6.96	599.67	196.36	−11.0	−10.3	−10.3	−9.7
	**e**	0	10	2	6.42	627.68	200.14	−11.5	−9.5	−10.7	−10.4
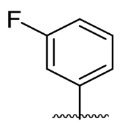 **9**	**a**	+1	8	4	4.81	523.57	170.03	−10.5	−10.3	−11.5	−9.8
	**b**	+1	9	2	5.44	606.71	196.15	−11.1	−9.1	−10.7	−9.3
	**c**	0	11	1	5.32	593.66	189.17	−11.3	−9.3	−10.6	−9.3
	**d**	0	9	2	6.96	599.67	196.36	−11.8	−10.3	−10.3	−10.3
	**e**	0	10	2	6.42	627.68	200.14	−11.7	−9.6	−10.7	−10.1
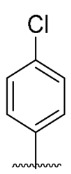 **10**	**a**	+1	8	4	5.23	540.02	174.62	−9.8	−10.3	−11.4	−9.6
	**b**	+1	9	2	5.86	623.16	200.74	−10.8	−9.1	−10.6	−9.6
	**c**	0	11	1	5.74	610.12	193.76	−10.7	−9.3	−10.5	−9.6
	**d**	0	9	2	7.38	616.12	200.95	−11.2	−10.2	−11.0	−10.0
	**e**	0	10	2	6.83	644.13	204.73	−11.1	−9.5	−10.6	−10.5
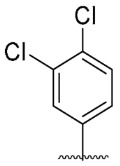 **11**	**a**	+1	8	4	5.79	574.47	179.42	−10.1	−10.4	−11.7	−9.9
	**b**	+1	9	2	6.42	657.60	205.54	−10.4	−9.3	−10.7	−9.0
	**c**	0	11	1	6.30	644.56	198.57	−11.0	−9.2	−10.7	−10.3
	**d**	0	9	2	7.94	650.56	205.76	−11.5	−10.7	−10.3	−9.3
	**e**	0	10	2	7.40	678.57	209.54	−11.4	−9.7	−10.6	−10.6
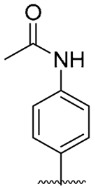 **12**	**a**	+1	10	5	3.99	562.63	184.68	−10.3	−10.1	−10.0	−9.6
	**b**	+1	11	3	4.62	645.77	210.80	−10.9	−9.9	−10.8	−10.2
	**c**	0	13	2	4.50	632.73	203.82	−10.8	−9.7	−10.6	−10.0
	**d**	0	11	3	6.14	638.73	211.01	−11.3	−10.6	−11.4	−9.8
	**e**	0	12	3	5.60	666.74	214.79	−11.2	−9.4	−10.7	−10.1
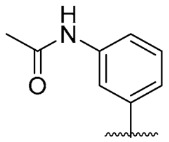 **13**	**a**	+1	10	5	3.99	562.63	184.68	−11.3	−10.6	−10.6	−10.4
	**b**	+1	11	3	4.62	645.77	210.80	−10.6	−9.5	−11.0	−9.4
	**c**	0	13	2	4.50	632.73	203.82	−10.7	−9.5	−10.4	−9.7
	**d**	0	11	3	6.14	638.73	211.01	−11.1	−10.4	−10.6	−9.7
	**e**	0	12	3	5.60	666.74	214.79	−11.2	−9.9	−10.9	−11.1
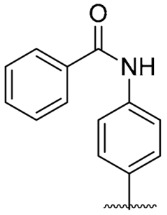 **14**	**a**	+1	10	5	5.72	624.71	205.35	−11.5	−10.1	−11.5	−9.9
	**b**	+1	11	3	6.35	707.84	231.47	−11.0	−9.5	−10.9	−10.0
	**c**	0	13	2	6.23	694.80	224.49	−10.8	−9.9	−10.5	−9.8
	**d**	0	11	3	7.87	700.80	231.68	−10.4	−10.4	−11.0	−10.2
	**e**	0	12	3	7.33	728.81	235.46	−11.0	−10.8	−11.1	−10.4
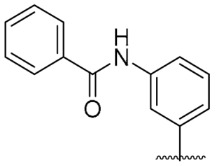 **15**	**a**	+1	10	5	5.72	624.71	205.35	−11.3	−10.0	−11.3	−9.8
	**b**	+1	11	3	6.35	707.84	231.47	−11.8	−10.4	−10.7	−11.0
	**c**	0	13	2	6.23	694.80	224.49	−11.8	−10.6	−10.5	−10.7
	**d**	0	11	3	7.87	700.80	231.68	−11.8	−10.5	−10.8	−9.9
	**e**	0	12	3	7.33	728.81	235.46	−12.4	−11.1	−11.1	−10.8
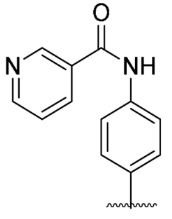 **16**	**a**	+1	11	5	4.49	625.69	203.19	−11.3	−10.1	−10.1	−9.8
	**b**	+1	12	3	5.12	708.83	229.31	−10.7	−10.2	−10.8	−9.6
	**c**	0	14	2	5.00	695.78	222.33	−10.5	−10.1	−10.4	−10.0
	**d**	0	12	3	6.64	701.79	229.52	−10.2	−10.9	−10.7	−10.0
	**e**	0	13	3	6.10	729.80	233.30	−10.5	−10.9	−11.2	−10.5
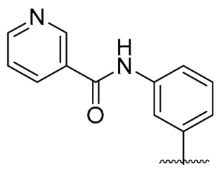 **17**	**a**	+1	11	5	4.49	625.69	203.19	−11.1	−10.2	−11.8	−10.6
	**b**	+1	12	3	5.12	708.83	229.31	−11.4	−9.8	−10.5	−9.8
	**c**	0	14	2	5.00	695.78	222.33	−11.5	−10.7	−10.6	−10.4
	**d**	0	12	3	6.64	701.79	229.52	−11.6	−10.7	−10.8	−10.3
	**e**	0	13	3	6.10	729.80	233.30	−12.3	−10.6	−11.1	−10.5
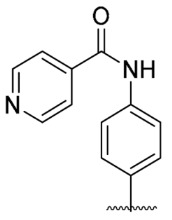 **18**	**a**	+1	11	5	4.49	625.69	203.19	−11.3	−10.1	−10.1	−9.8
	**b**	+1	12	3	5.12	708.83	229.31	−10.6	−8.4	−10.4	−10.4
	**c**	0	14	2	5.00	695.78	222.33	−10.4	−10.0	−10.4	−9.8
	**d**	0	12	3	6.64	701.79	229.52	−10.3	−9.8	−10.6	−9.7
	**e**	0	13	3	6.10	729.80	233.30	−10.8	−10.8	−10.9	−10.3
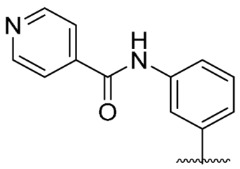 **19**	**a**	+1	11	5	4.49	625.69	203.19	−11.4	−9.4	−10.3	−10.6
	**b**	+1	12	3	5.12	708.83	229.31	−11.6	−10.6	−10.5	−10.7
	**c**	0	14	2	5.00	695.78	222.33	−11.5	−10.5	−10.6	−9.8
	**d**	0	12	3	6.64	701.79	229.52	−11.6	−11.2	−10.8	−9.6
	**e**	0	13	3	6.10	729.80	233.30	−12.2	−10.5	−11.2	−10.8
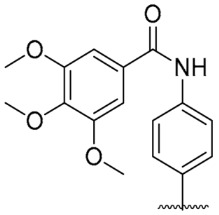 **20**	**a**	+1	16	5	5.14	714.78	224.74	−10.2	−9.5	−10.3	−9.7
	**b**	+1	17	3	5.76	797.92	250.86	−10.9	−8.2	−10.3	−9.1
	**c**	0	19	2	5.64	784.87	243.88	−10.9	−9.6	−10.1	−8.9
	**d**	0	17	3	7.29	790.89	251.07	−10.7	−10.4	−9.4	−9.0
	**e**	0	18	3	6.74	818.89	254.85	−11.1	−10.3	−10.8	−9.7
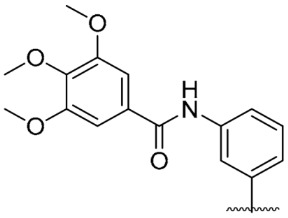 **21**	**a**	+1	16	5	5.14	714.78	224.74	−10.8	−9.8	−10.6	−10.1
	**b**	+1	17	3	5.76	797.92	250.86	−10.9	−9.1	−11.1	−8.9
	**c**	0	19	2	5.64	784.87	243.88	−10.8	−9.0	−11.0	−9.1
	**d**	0	17	3	7.29	790.89	251.07	−11.5	−9.6	−10.8	−8.8
	**e**	0	18	3	6.74	818.89	254.85	−11.5	−10.5	−11.3	−10.7

## Data Availability

Docking files are available upon request.

## Supplementary Materials

The following supporting information can be downloaded at: https://www.mdpi.com/article/10.3390/polym15071703/s1, Table S1: Grid Box centre axial coordinates (x,y,z) for the four targets under study, Table S2: ΔG_binding_ of synthetic precursors **25** for the four targets under study, Table S3: General procedure for the synthesis of starting reagents **23**, Table S4: ^1^H NMR (DMSO-*d*_6_, 400 MHz) and ^13^C NMR (DMSO-*d*_6_, 100 MHz) spectra of representative synthesised compounds, Table S5: MS spectra of representative synthesised inhibitors, Figure S1: Interaction-binding mode of docked ligands in the active site of PI3Kα.
