# Supplementary material for: Synthesis of Novel 2,9-Disubstituted-6-morpholino Purine Derivatives Assisted by Virtual Screening and Modelling of Class I PI3K Isoforms
Source: Polymers (Basel). 2023 Mar 29;15(7):1703. doi: 10.3390/polym15071703 (PMC10096987; doi:10.3390/polym15071703)

# Synthesis of novel 2,9-disubstituted-6-morpholino purine derivatives assisted by Virtual Screening and modelling of Class I PI3K isoforms

Vítor Lobo [1], Ashly Rocha [1], Tarsila G. Castro [2,3,*] and Maria Alice Carvalho [1,*]

1Chemistry Centre, School of Sciences, University of Minho, 4710-057 Braga, Portugal;

[2]CEB -Center of Biological Engineering, University of Minho, Campus de Gualtar, 4710-057, Braga, Portugal;

[3]LABBELS—Associate Laboratory, Campus de Gualtar, 4710-057Braga, Portugal.

# Supporting Information

**Table S1.** Grid Box centre axial coordinates (x,y,z) for the 4 targets under study.

**Table S2**. $\Delta G_{binding}$ of synthetic precursors **25** for the 4 targets under study.

**Figure S3.** Interaction-binding mode of docked ligands in the active site of PI3Kα.

**Table S4**. General procedure for the synthesis of starting reagents 23

**Table S5.** $^{1}$H NMR (DMSO-d6, 400 MHz) and $^{13}$C NMR (DMSO-d6, 100 MHz) spectra of representative synthesised compounds.

**Table S6** MS spectra of representative synthesised inhibitors

**Table S1.** Grid Box centre axial coordinates (x,y,z) for the 4 targets under study.

| | PI3Kα | PI3Kβ | PI3Kγ | PI3Kδ |
|---|---|---|---|---|
| **x center** | -18.53 | -26.22 | 17.04 | -8.60 |
| **y center** | 11.64 | 21.24 | -5.37 | -30.25 |
| **z center** | 25.60 | 27.38 | 18.23 | 23.87 |

**Table S2.** $\Delta G_{binding}$ of synthetic precursors **25** for the 4 targets under study.

| **R1** | $\Delta G_{binding}$ (kcal/mol) | | | |
|---|---|---|---|---|
| | **PI3Kα** | **PI3Kβ** | **PI3Kγ** | **PI3Kδ** |
| 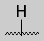 | -8.6 | -8.4 | -8.3 | -8.0 |
| 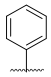 | -10.0 | -8.3 | -9.1 | -8.8 |
| 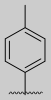 | -10.0 | -8.5 | -9.3 | -8.7 |
| 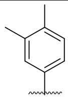 | -10.4 | -8.7 | -9.8 | -8.9 |
| 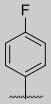 | -9.8 | -8.4 | -9.2 | -9.2 |
| 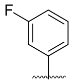 | -10.2 | -8.5 | -9.3 | -9.2 |

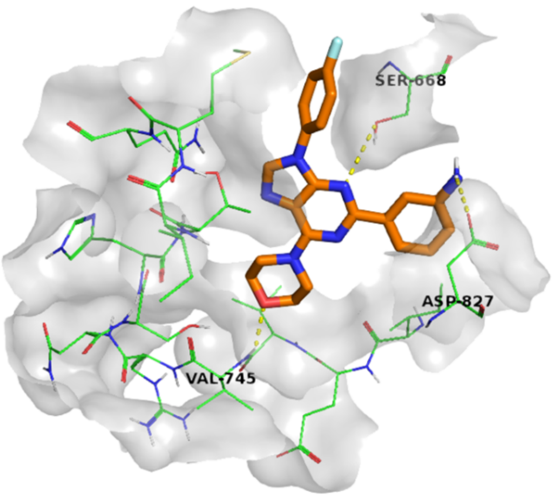


**Figure S3.** Interaction-binding mode of docked ligands in the active site of PI3Kα.

**Table S4**. General procedure for the synthesis of starting reagents 23

Morpholine (5 equivalent) was added to a suspension of 5-amino-4-cyanoformimidoylimidazole **22a-h** in acetonitrile. The mixture was stirred at room temperature until all the starting material was consumed (evidence by TLC). The obtained solid that precipitated from the reaction mixture was filtered and washed with acetonitrile and diethyl ether.

*2.2.2.1. 4-[Imino(morpholin-4-yl)methyl]-1-(3,4-dimethylphenyl)-1H-imidazol-5-amine (23e)*

Compound **23e** (4.84 g, 16.17 mmol, 87%), was obtained as a grey solid from the reaction of [5-amino-1-(3,4-dimethylphenyl)-1H-imidazol-4-yl](imino)acetonitrile **22e** (4.43 g, 18.49 mmol) with morpholine (6.45 mL, 73.97 mmol, 4 eq.) in acetonitrile (30 mL) after 21 hours. M.p. 175 – 177 °C. IR (nujol) νmax: 3377, 3119, 1624, 1595, 1586, 1548, 1515 $cm^{-1}$. $^1H$ NMR (400 MHz, DMSO-$d_6$) $\delta$: 7.31 (s, 1H), 7.29 (d, 1H, $J^3$ = 8.0 Hz), 7.25 (t, 1H, $J^4$ = 2.0 Hz), 7.17 (dd, 1H, $J^3$ = 8.0 Hz, $J^4$ = 2.0 Hz), 6.7 – 4.7 (br.s, 2H, $NH_2$, exchangeable by $D_2O$), 3.67 (t, 4H, $J^3$ = 4.8 Hz), 3.33 (t, 4H, $J^3$ = 4.8 Hz), 2.27 (s, 3H), 2.26 (s, 3H); $^{13}C$ NMR (100 MHz, DMSO-$d_6$) $\delta$: 163.23, 139.41, 137.90, 136.33, 132.45, 130.47, 129.86, 125.66, 122.02, 114.27, 66.07, 47.13, 19.33, 18.93. MS (ESI) 300 [$M^+$ + 1]. HRMS (ESI): *m*/*z* [$M^+$ + 1] calcd for $C_{16}H_{22}N_5O$: 300.1824; Found: 300.1829.

*2.2.2.2. 4-[Imino(morpholin-4-yl)methyl]-1-(4-aminophenyl)-1H-imidazol-5-amine (23h)*

Compound **23h** (3.64 g, 12.72 mmol, 95%), was obtained as an off-white solid from the reaction of [5-amino-1-(4-aminophenyl)-1H-imidazol-4-yl](imino)acetonitrile **22h** (3.04 g, 13.43 mmol) with morpholine (5.86 mL, 67.14 mmol, 5 eq.) in acetonitrile (23 mL) after 16 hours. M.p. 178 – 180 °C. IR (nujol) νmax: 3322, 3288, 3196, 3113, 1626, 1609, 1586, 1550, 1521 $cm^{-1}$. $^1H$ NMR (400 MHz, DMSO-$d_6$) $\delta$: 7.18 (s, 1H), 7.04 (d, 2H, $J^3$ = 8.4 Hz), 6.66 (d, 2H, $J^3$ = 8.4 Hz), 6.0 – 4.7 (br s, 4H, $NH_2$, exchangeable by $D_2O$), 3.66 (t, 4H, $J^3$ = 4.4 Hz), 3.30 (t, 4H, $J^3$ = 4.4 Hz); $^{13}C$ NMR (100 MHz, DMSO-$d_6$) $\delta$: 163.55, 148.99, 139.51, 130.31, 126.13, 122.66, 114.10, 113.76, 66.12, 47.25. MS (ESI) 287 [$M^+$ + 1]. HRMS (ESI): *m*/*z* [$M^+$ + 1] calcd for $C_{14}H_{19}N_6O$: 287.1620; Found: 287.1625.

**Table S5.** $^{1}H$ NMR (DMSO-d6, 400 MHz) and $^{13}C$ NMR (DMSO-d6, 100 MHz) spectra of representative synthesised compounds.

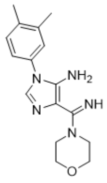

**23e**

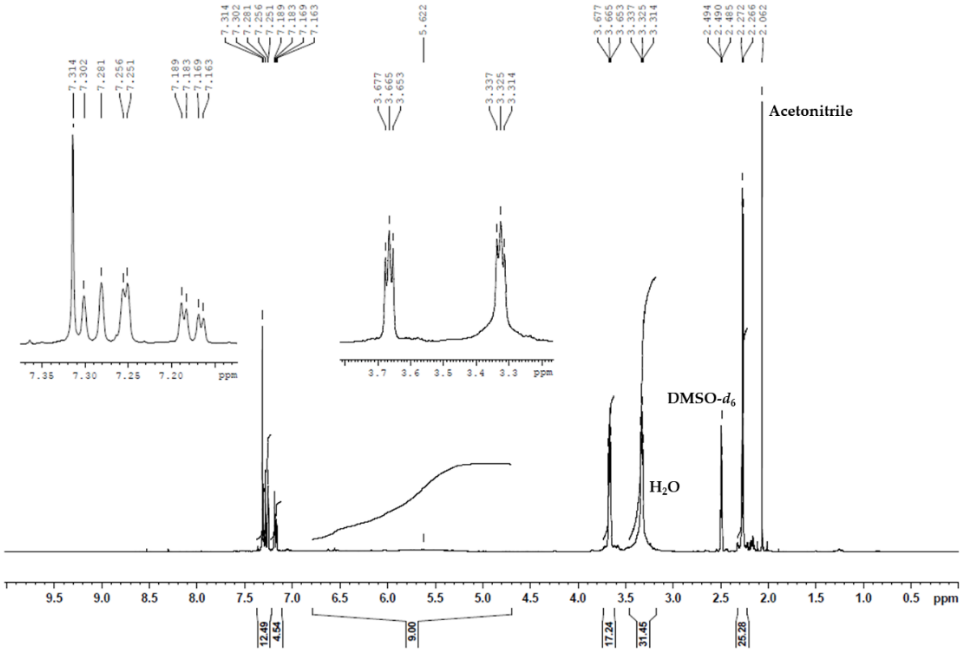


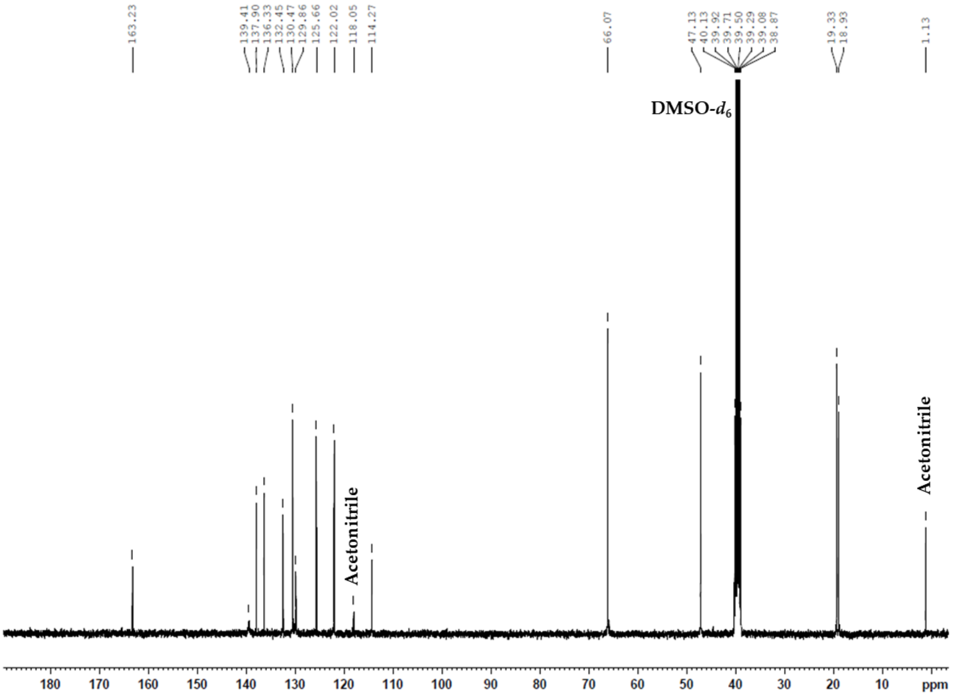

**Table S5.** cont.

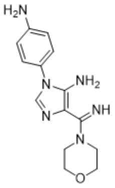

**23h**

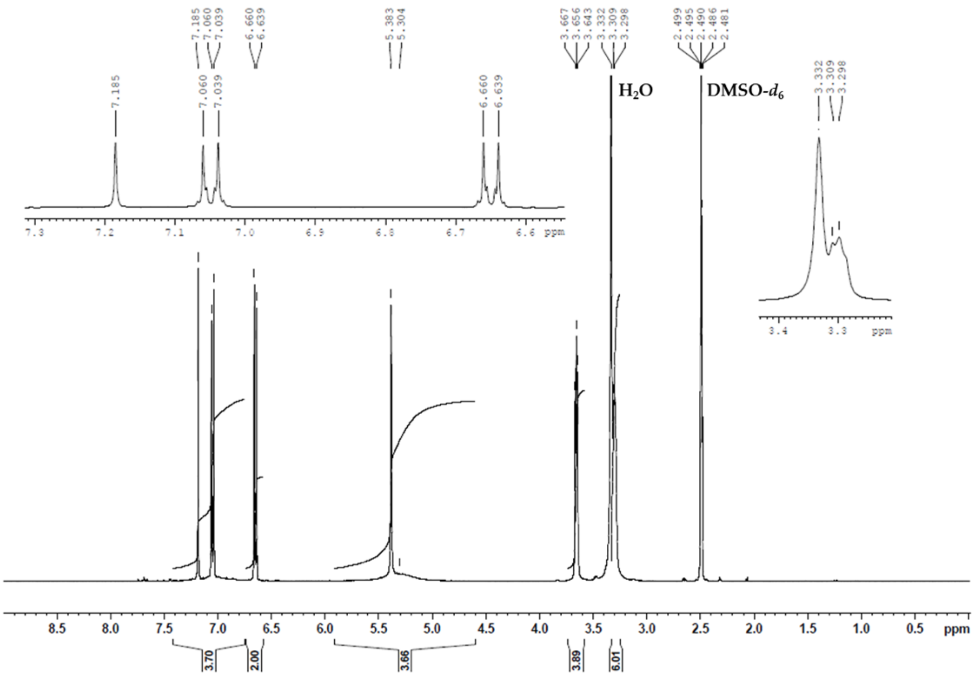


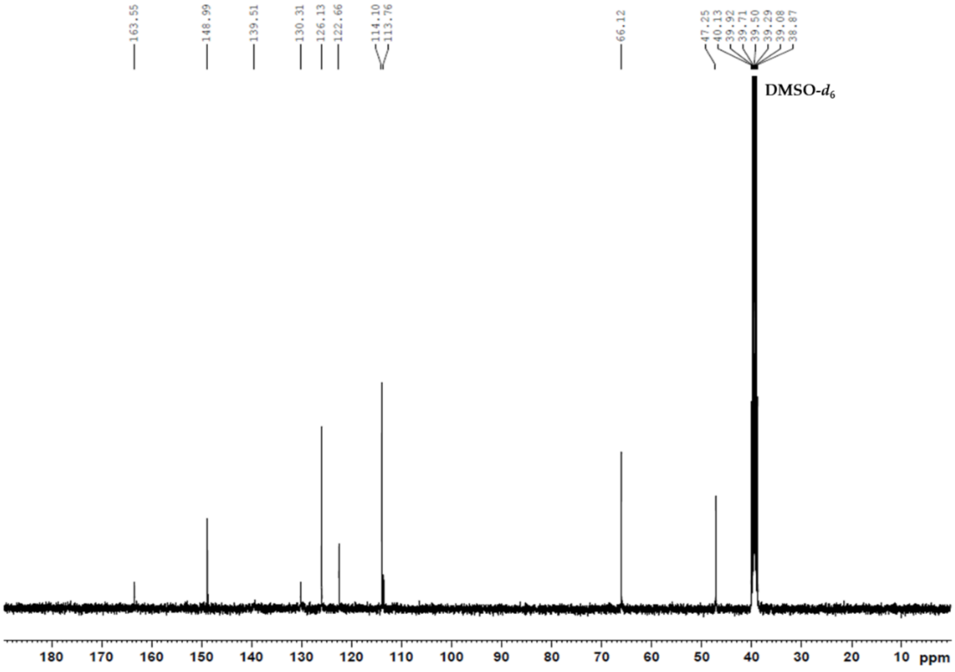

**Table S5.** cont.

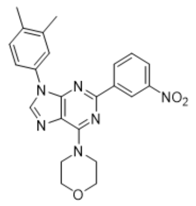

**24e**

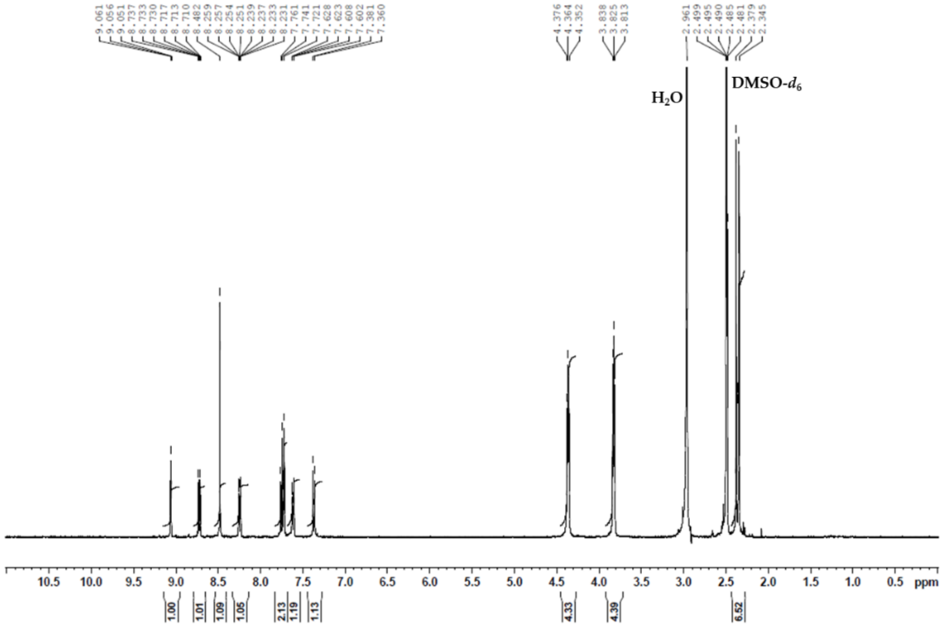


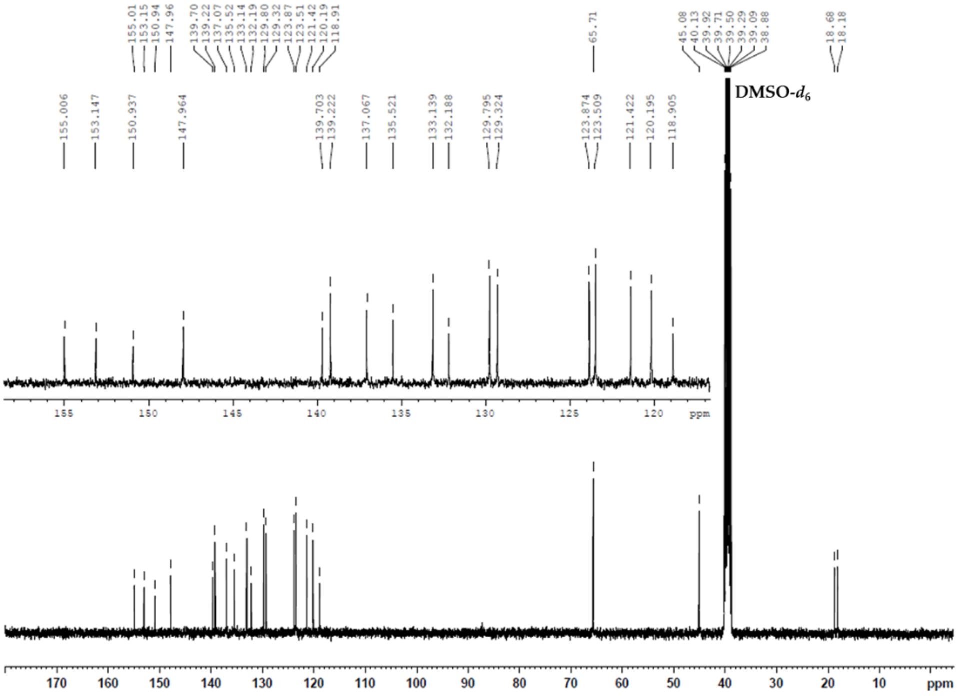

**Table S5.** cont.

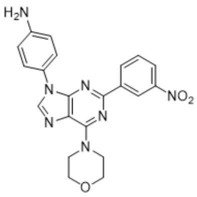


**24h**

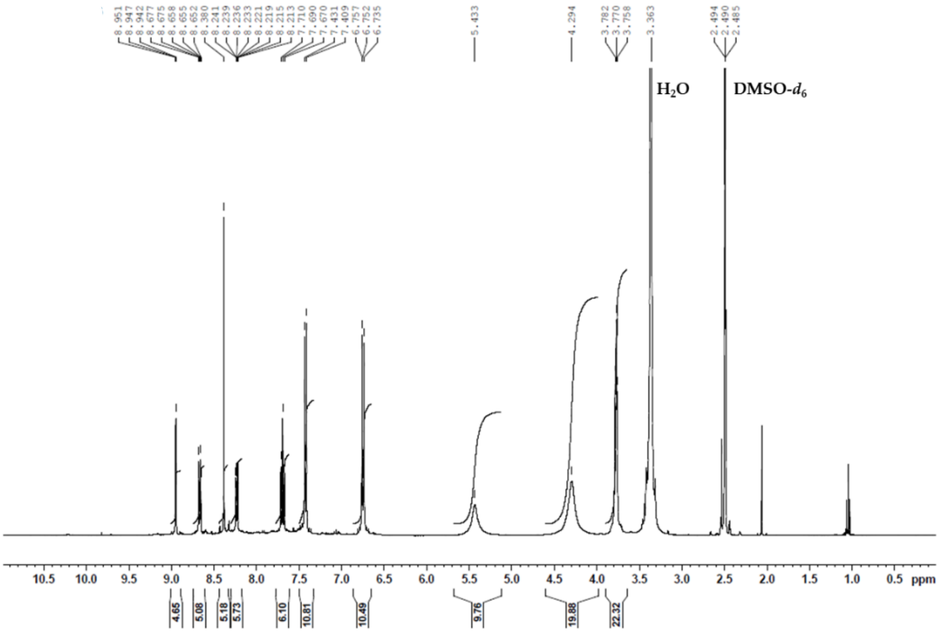


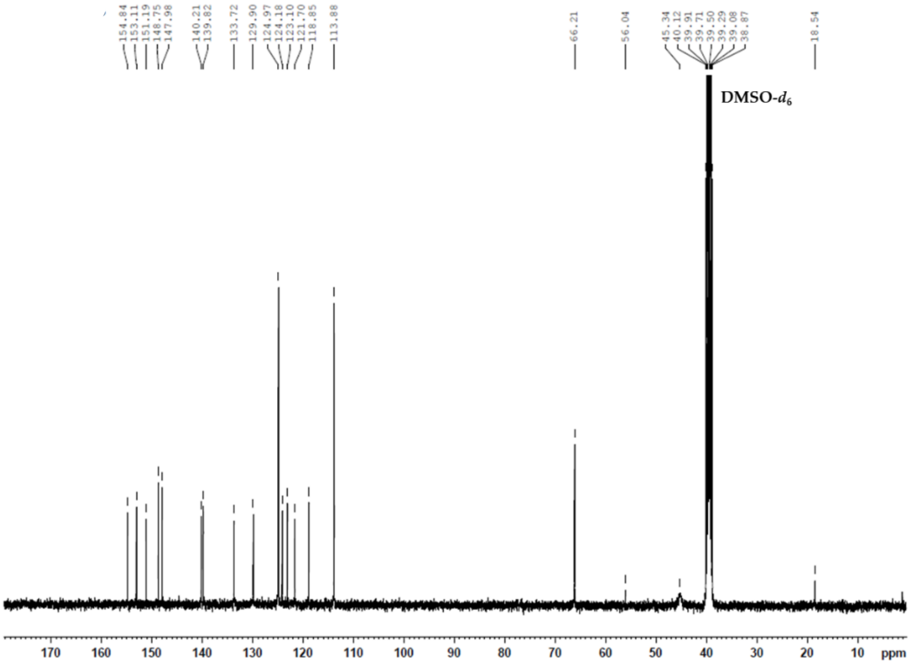

**Table S5.** cont.

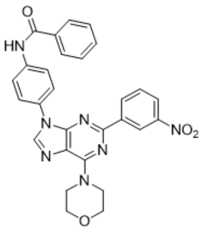


**24i**

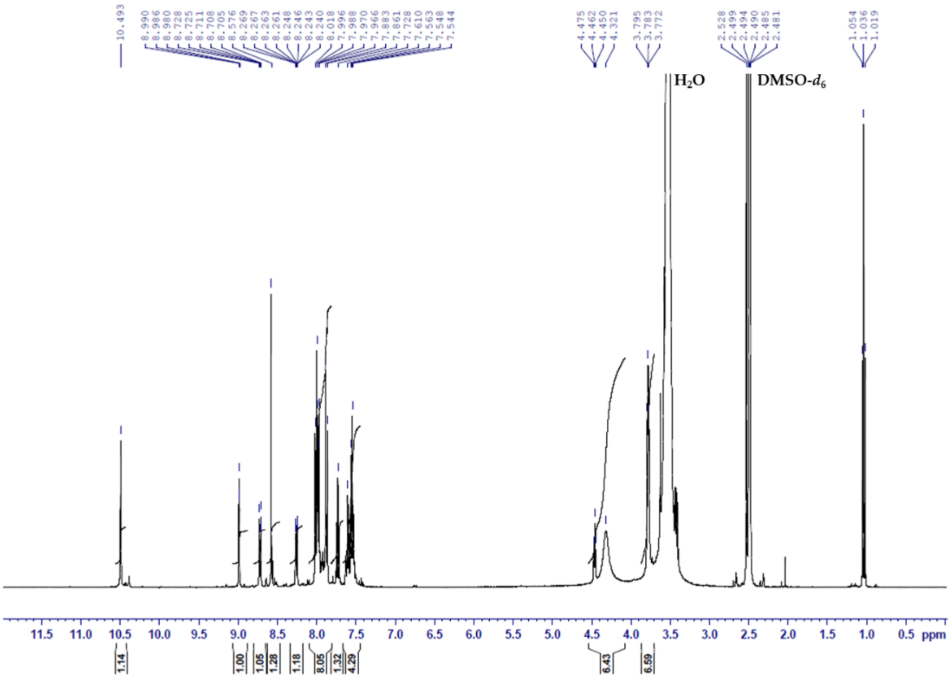


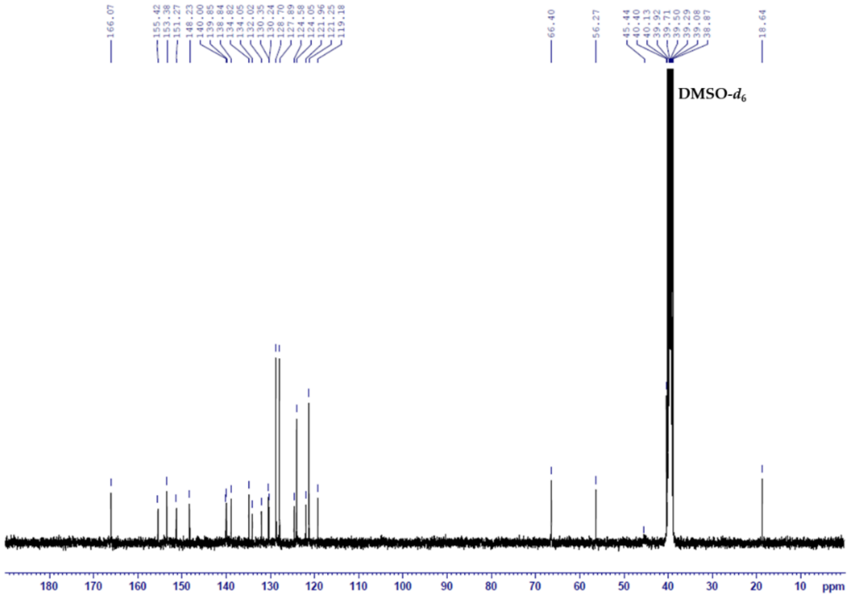

**Table S5.** cont.

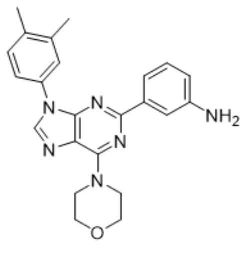


**25e**

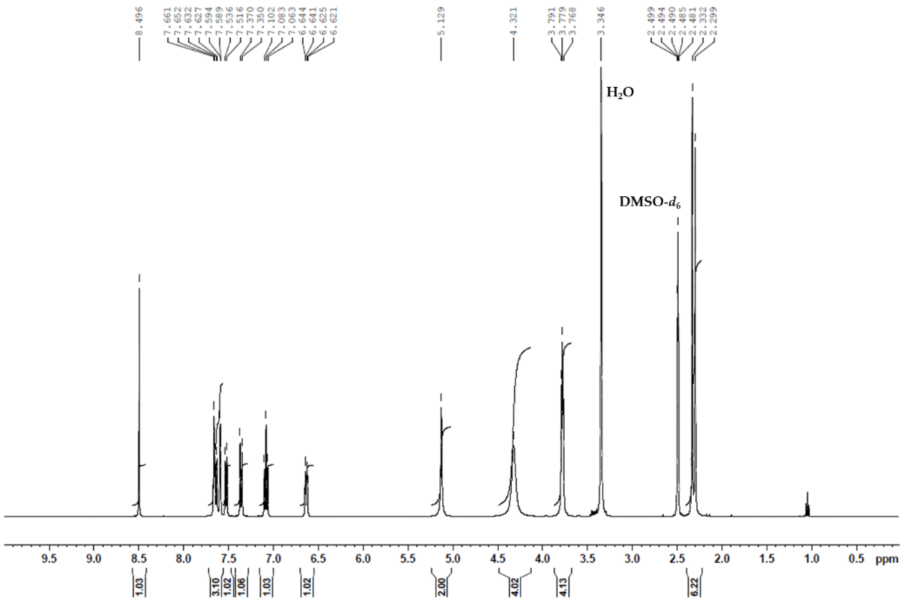


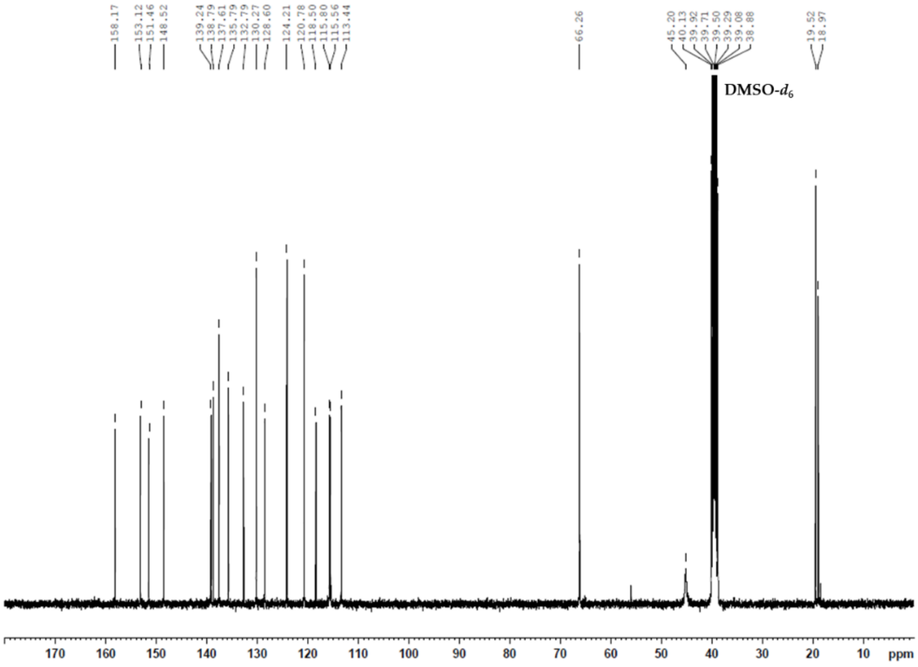

**Table S5.** cont.

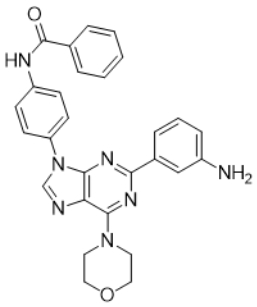

**25h**

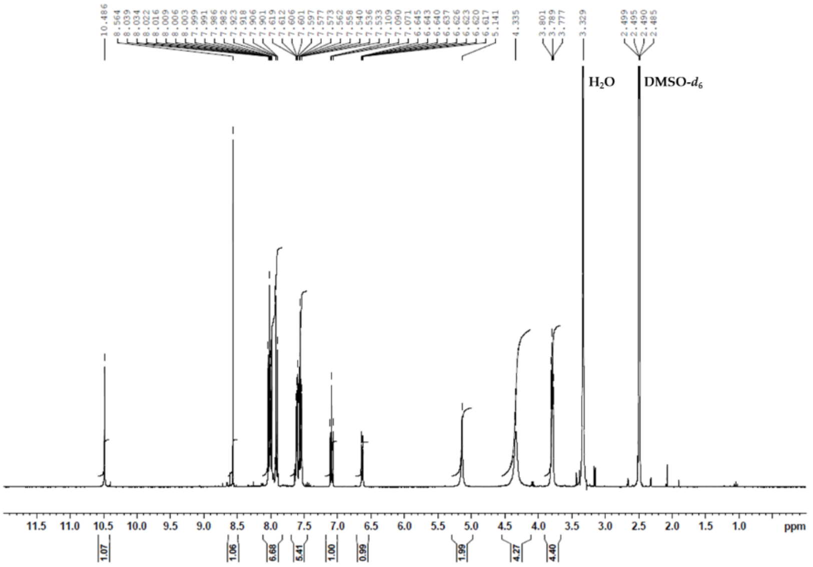


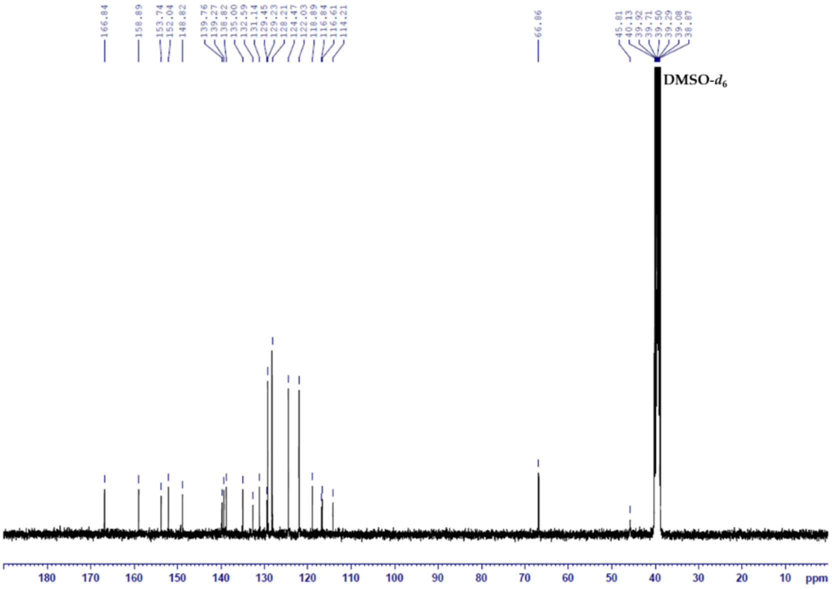

**Table S5.** cont.

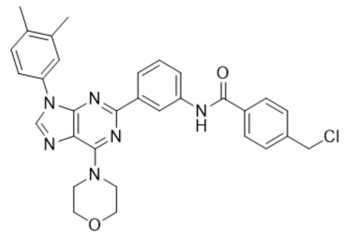

**26e**

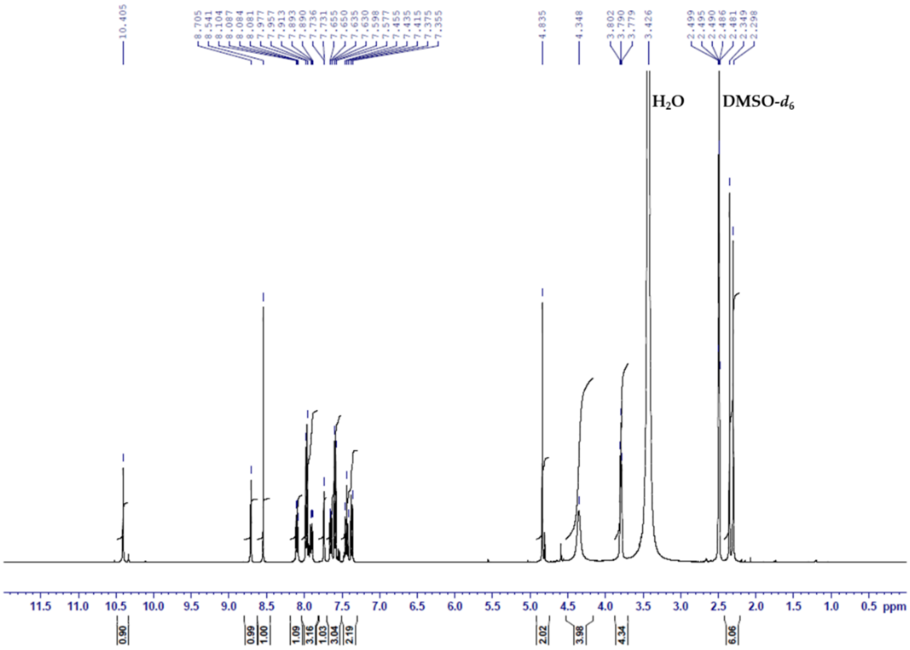


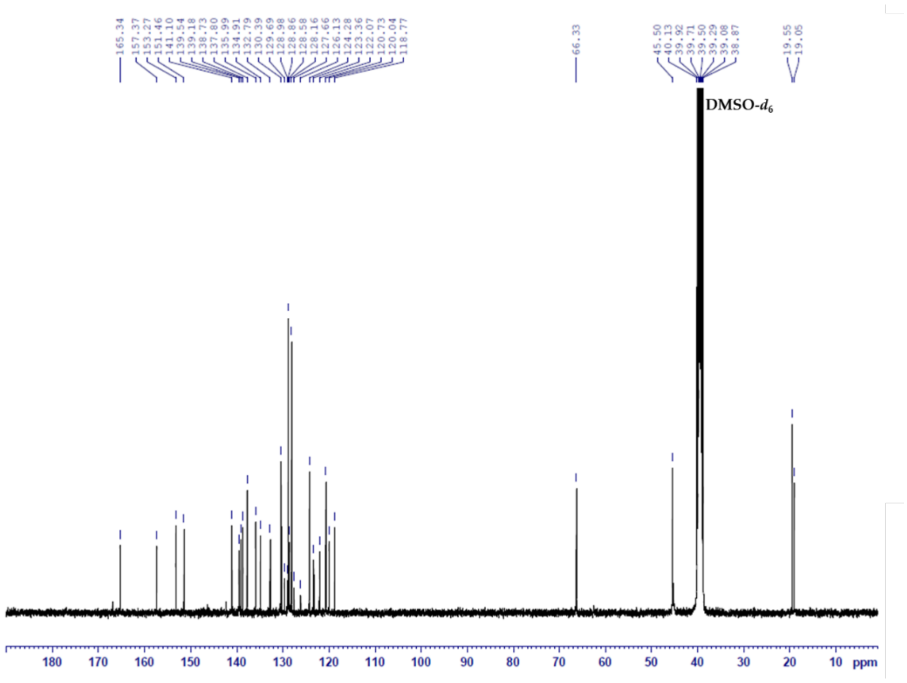

**Table S5.** cont.

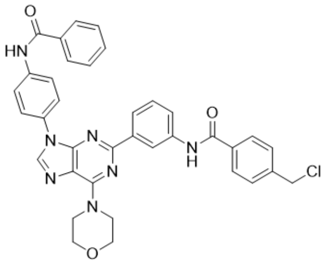


**26h**

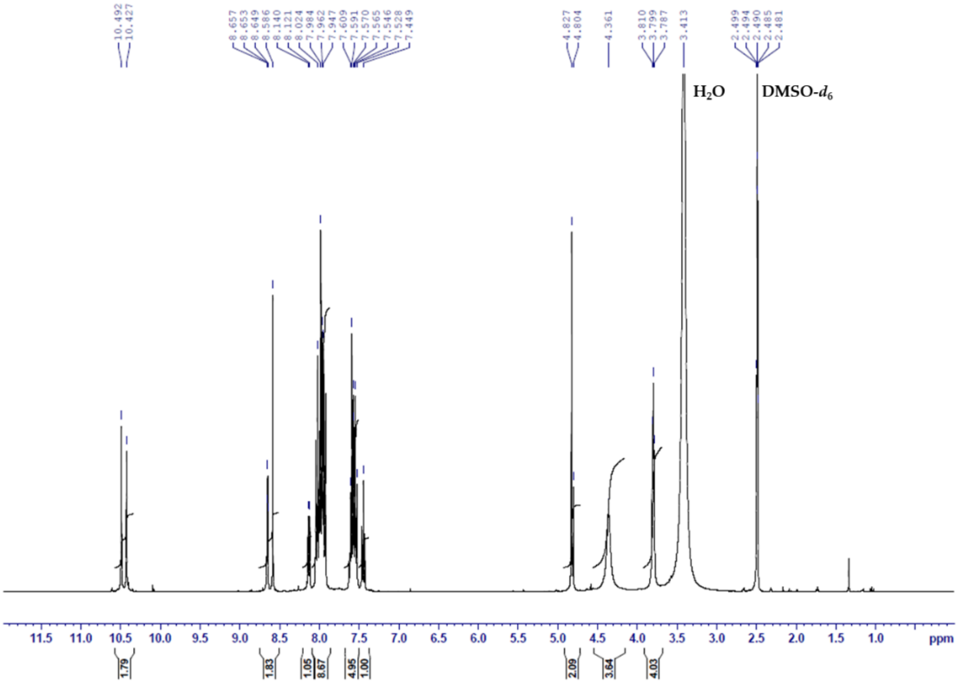


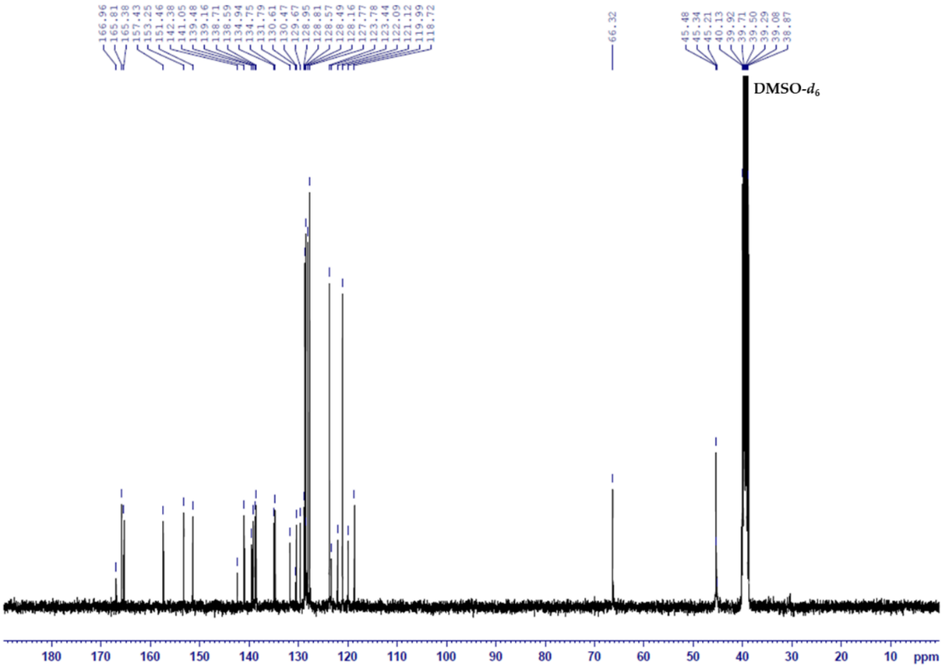

**Table S5.** cont.

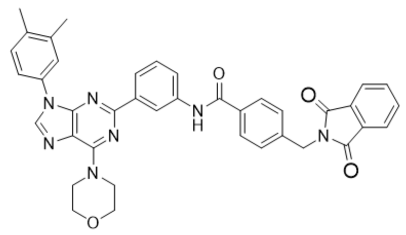

**27**

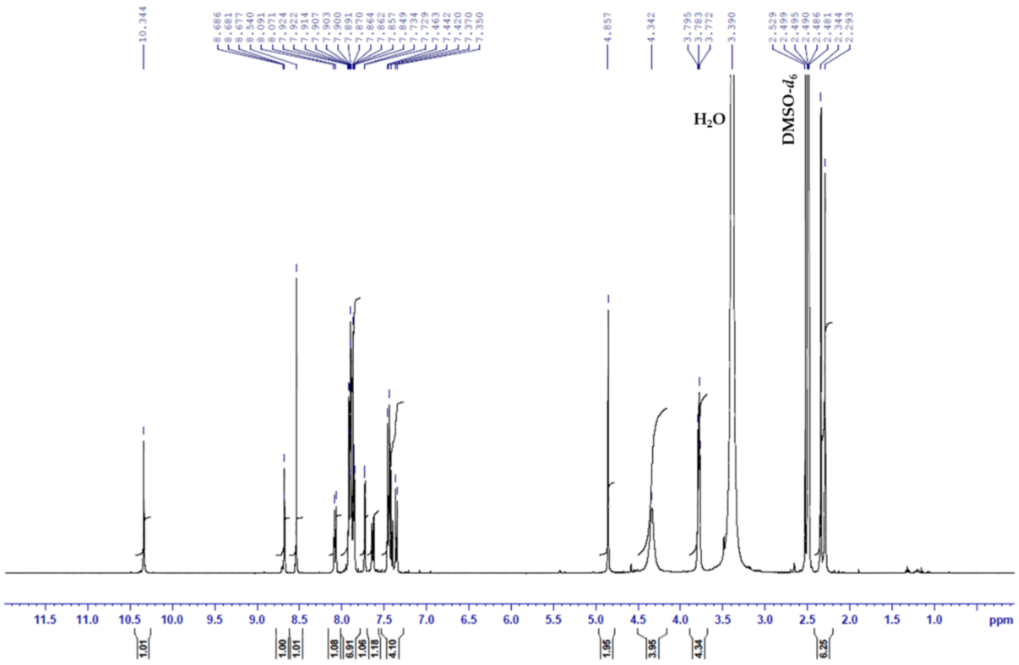

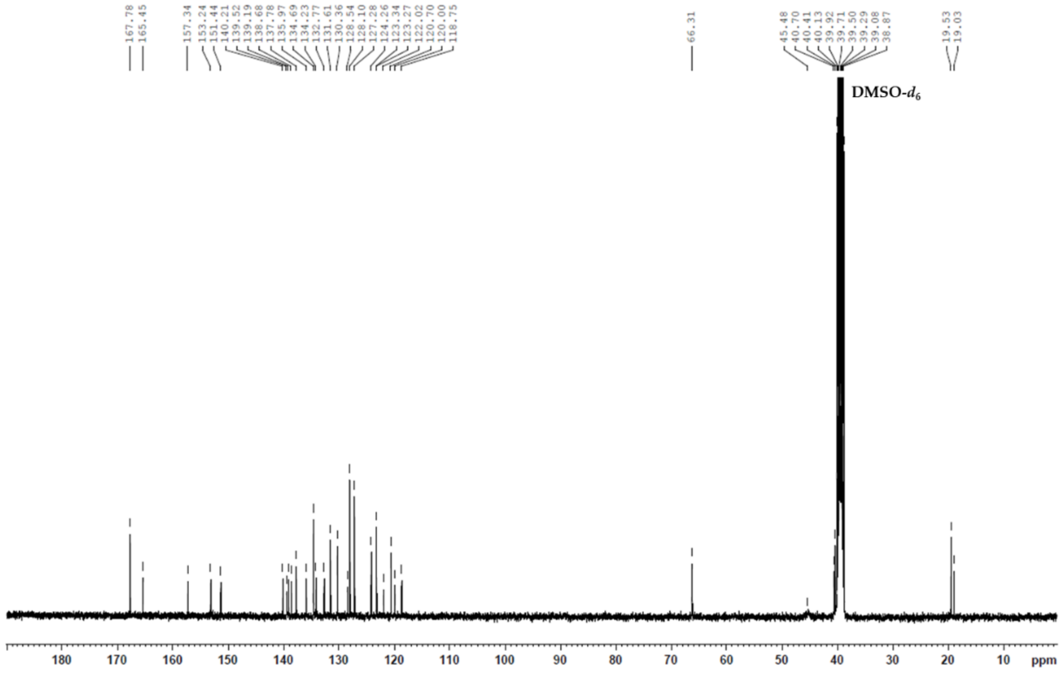

**Table S5.** cont.

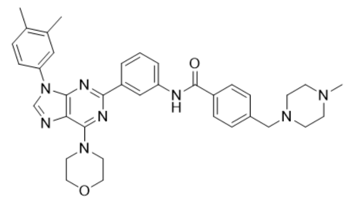

**7b**

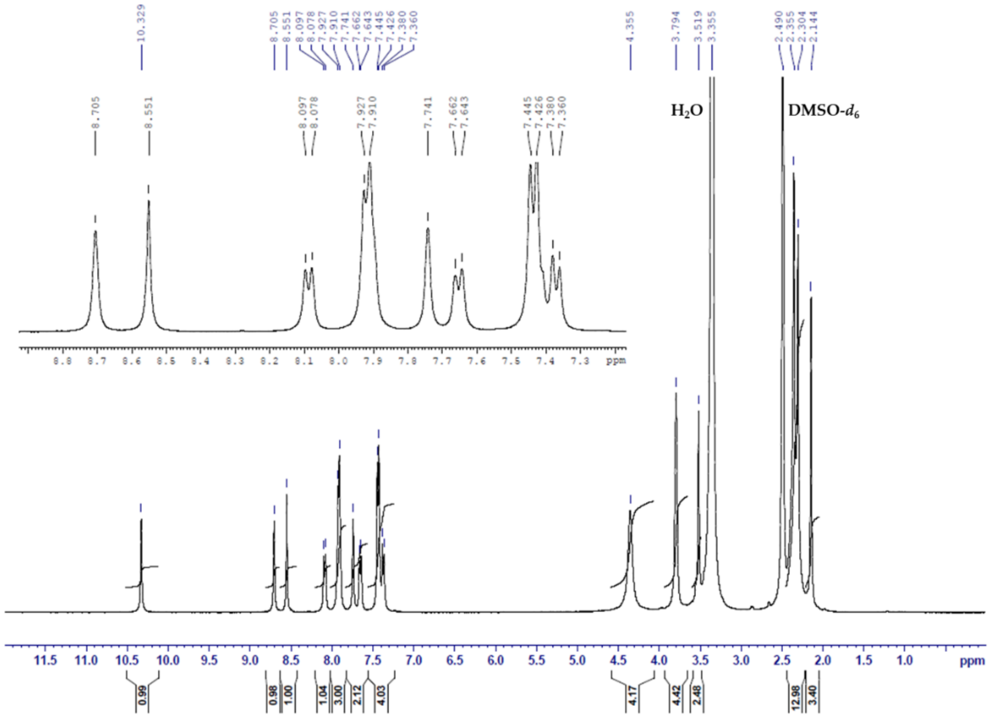


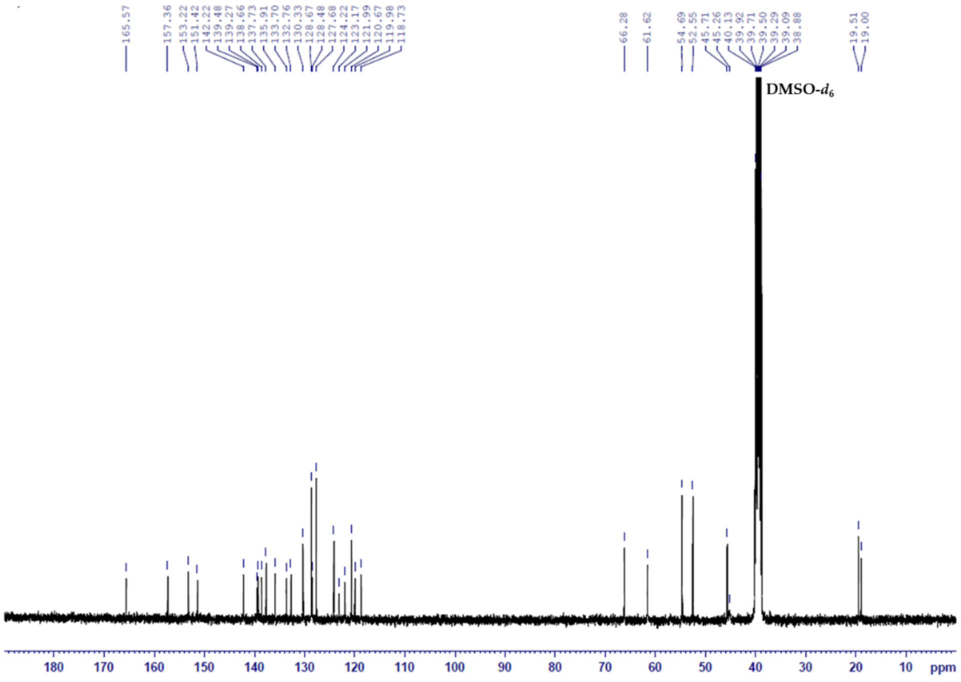

**Table S5.** cont.

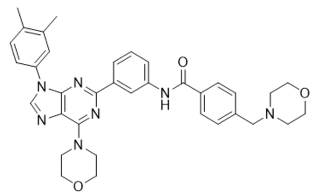

**7c**

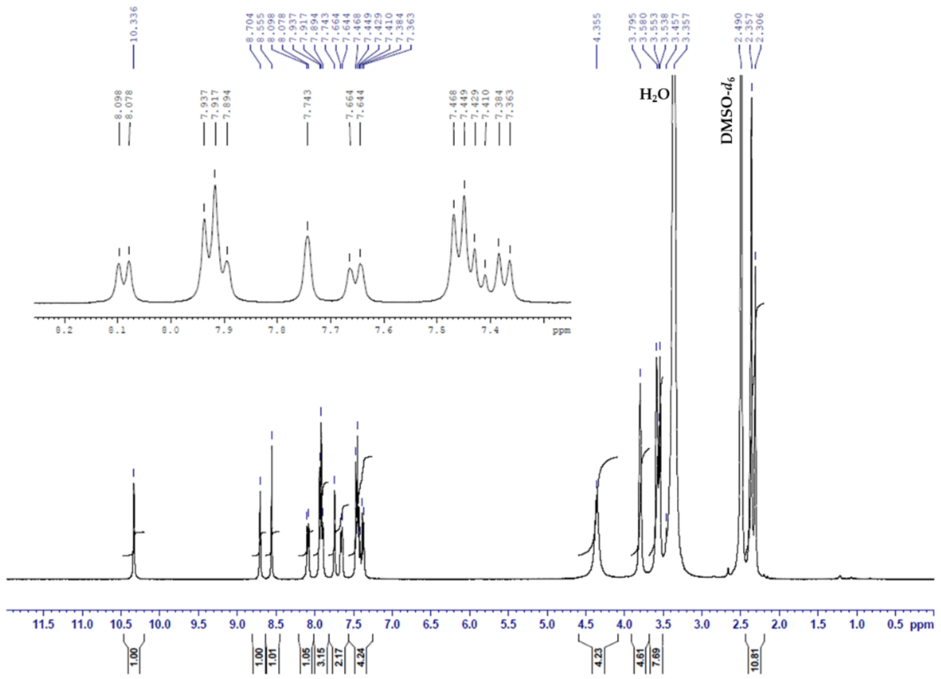


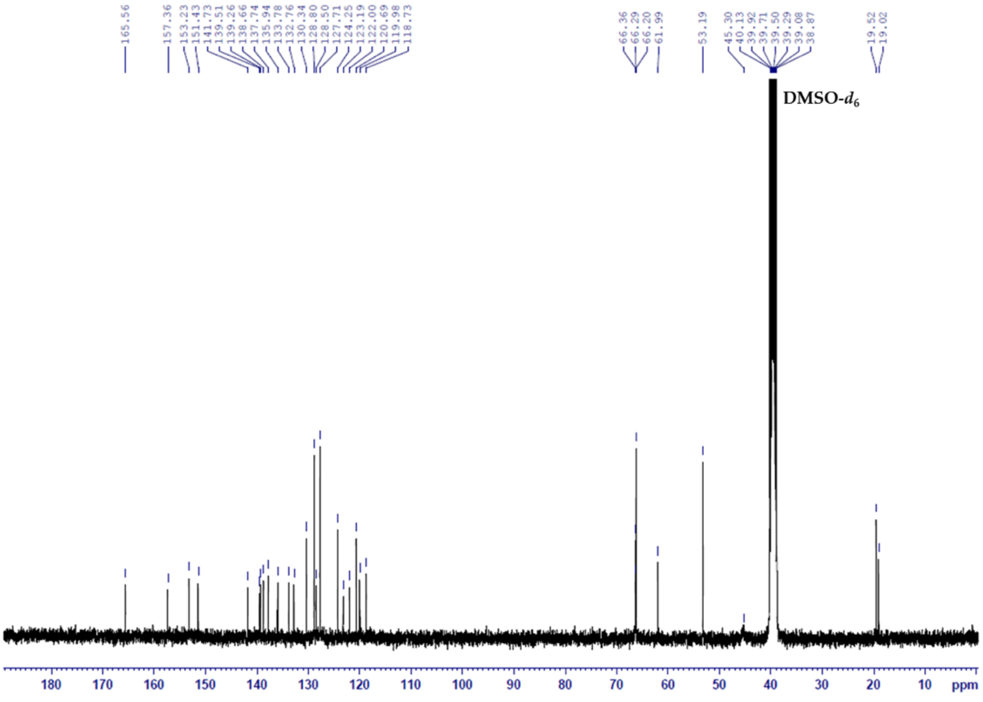

**Table S5.** cont.

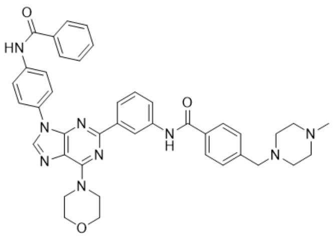

**14b**

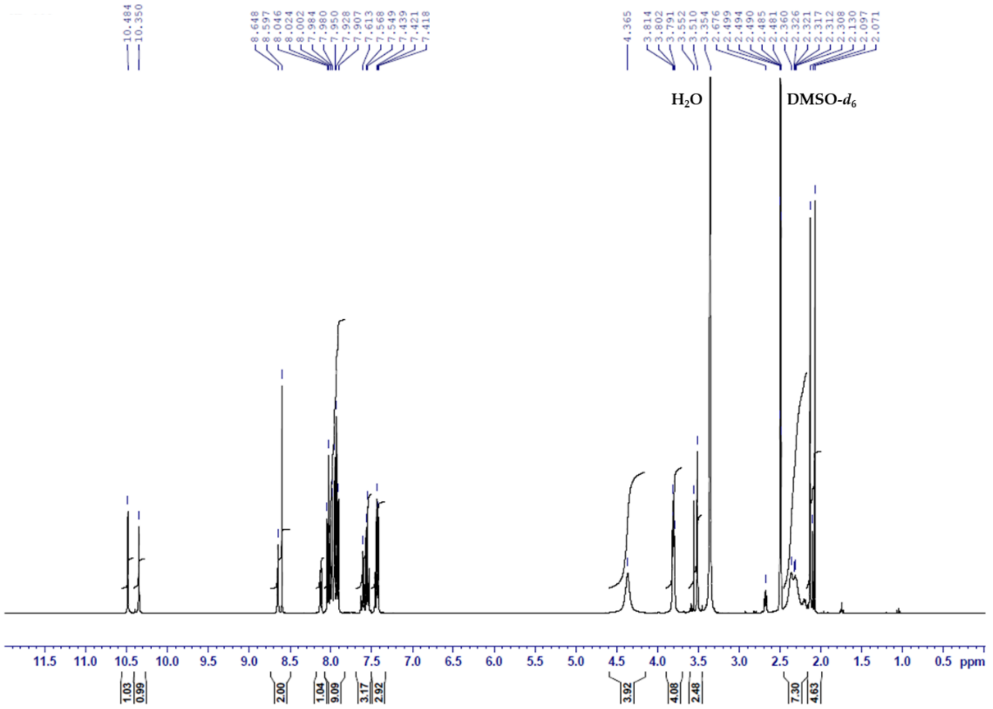


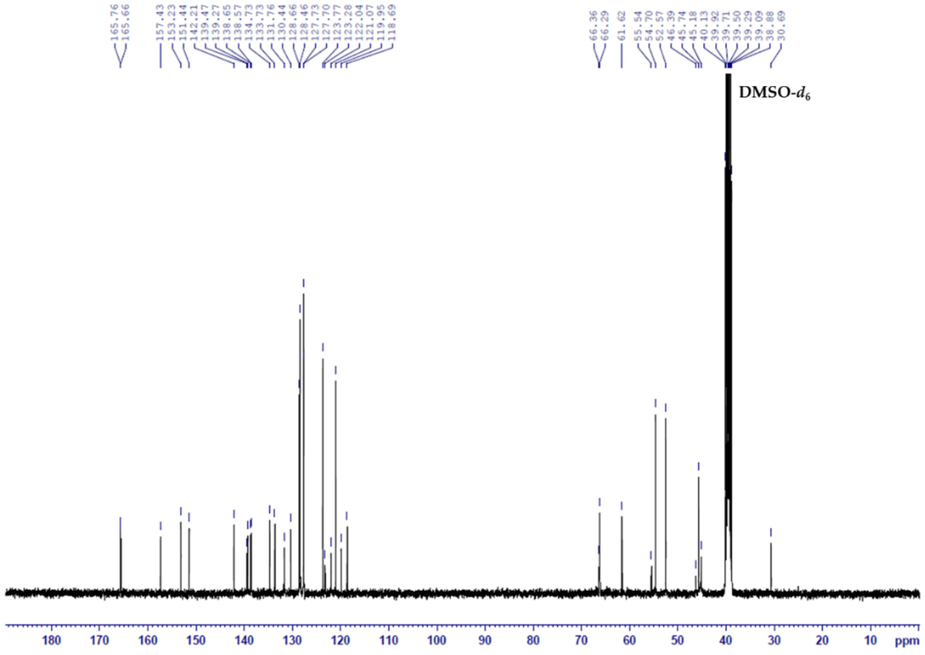

**Table S5.** cont.

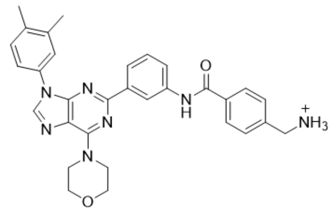


**7a**

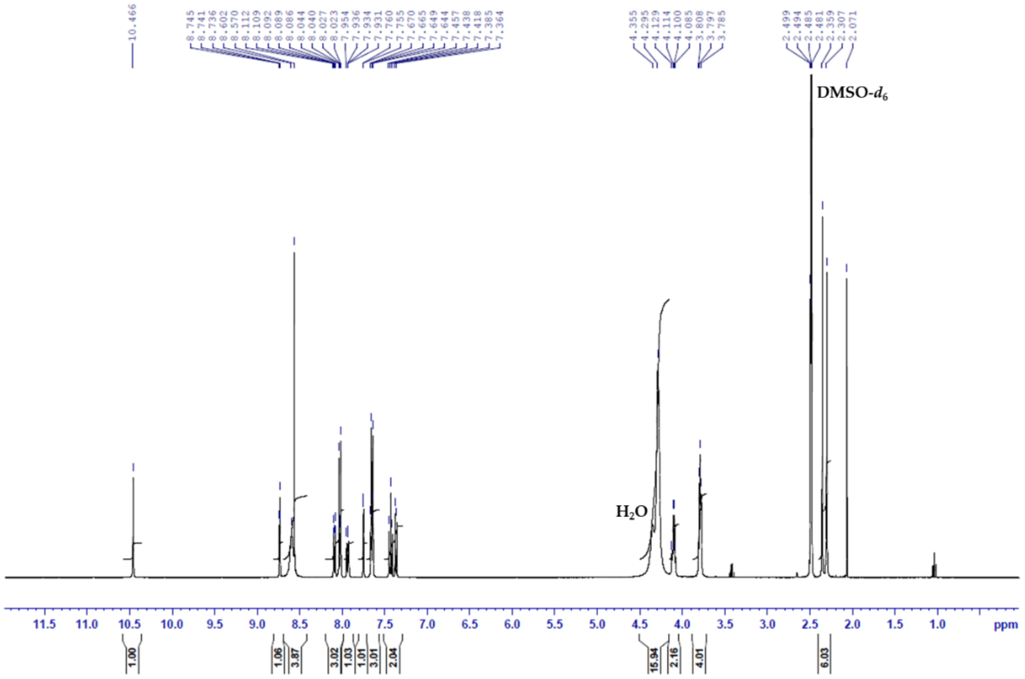


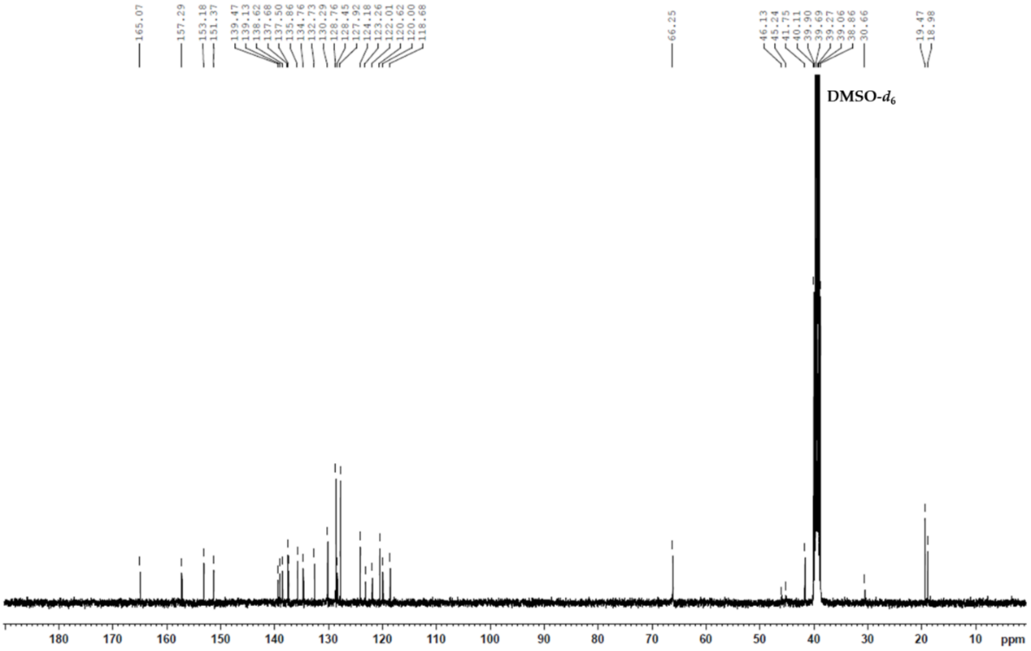

**Table S5.** cont.

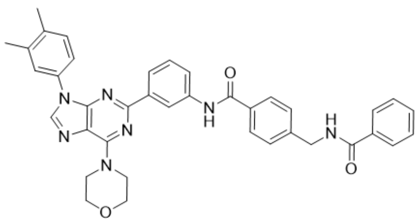

**7e**

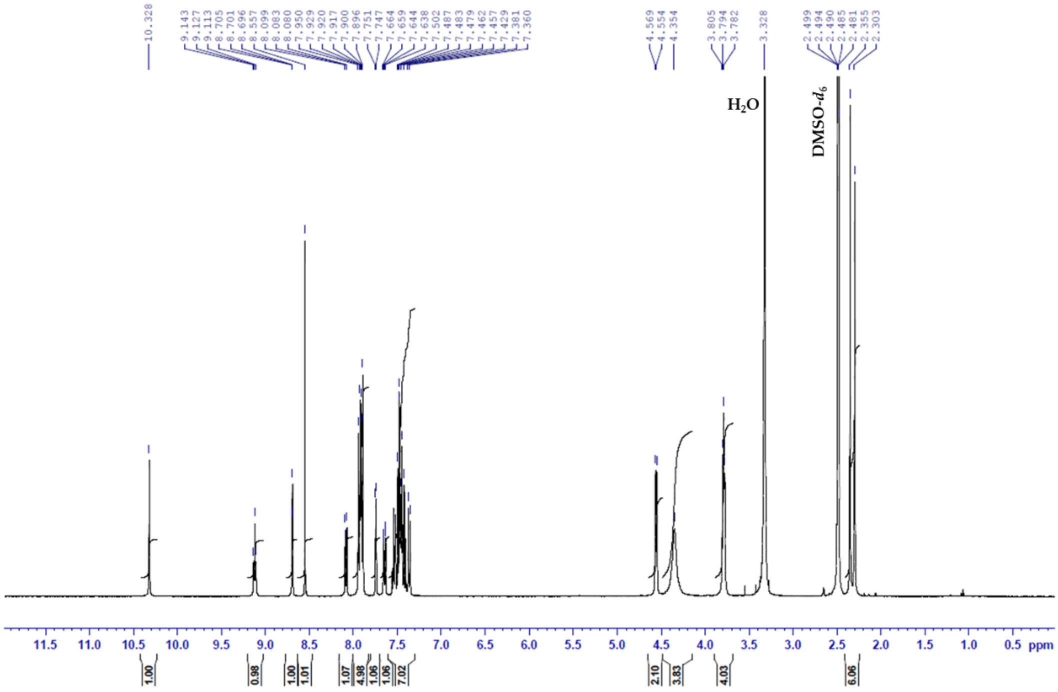


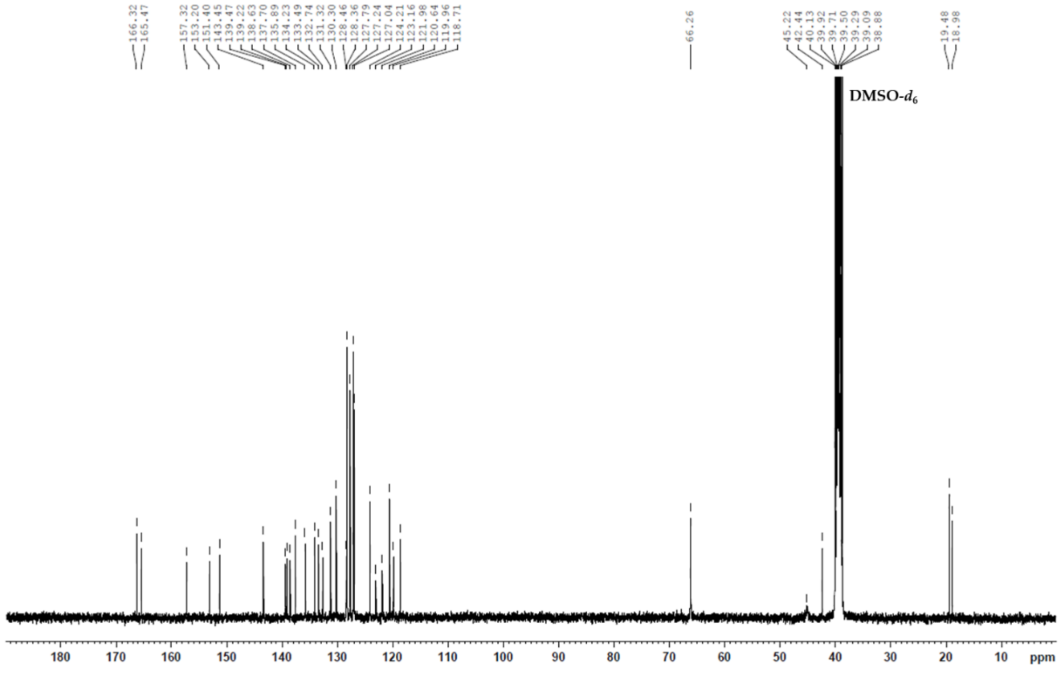

**Table S6.** MS spectra of representative synthesised inhibitors

## Compound 7a

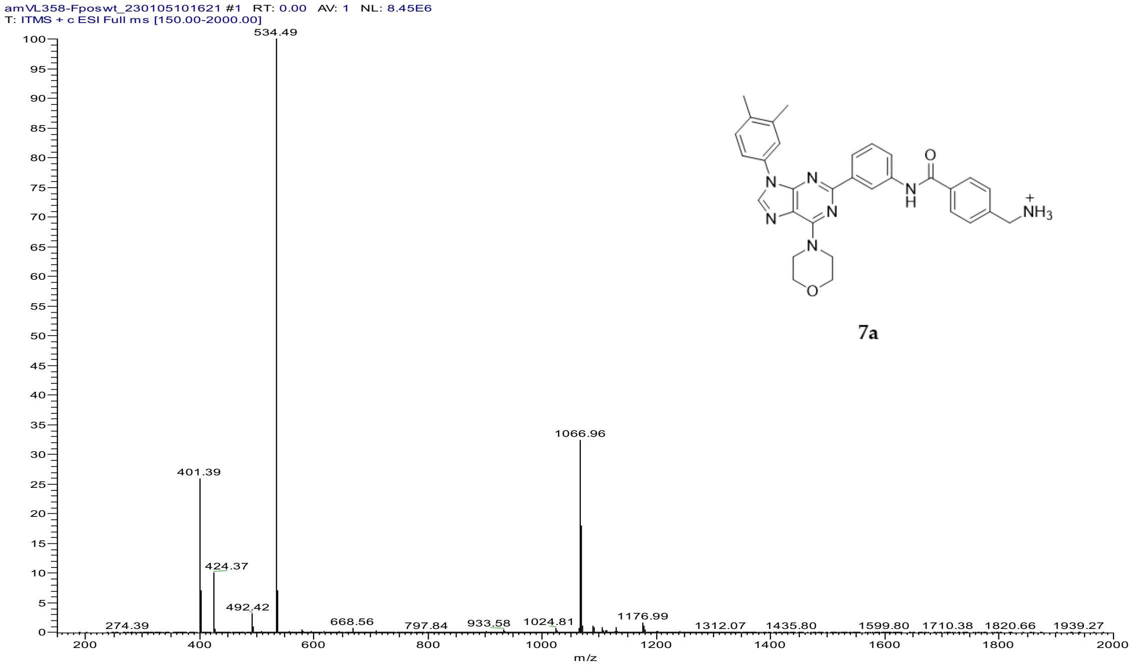


## Compound 7b

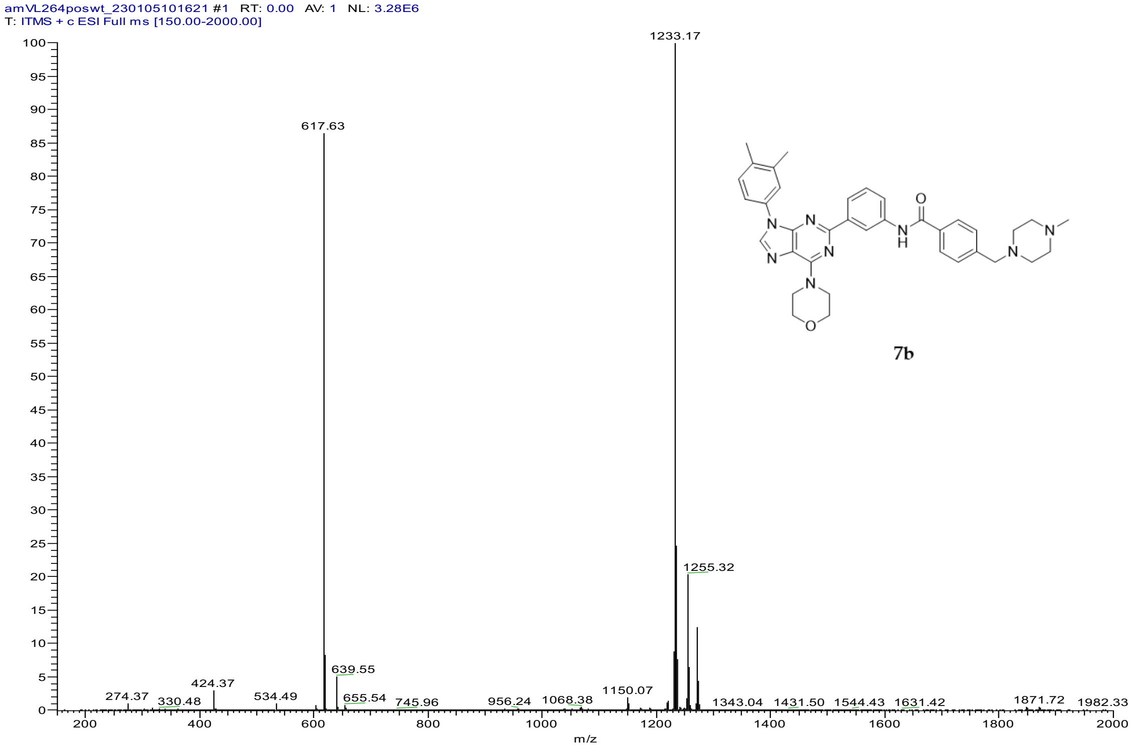

**Table S6.** cont.

## Compound 7c

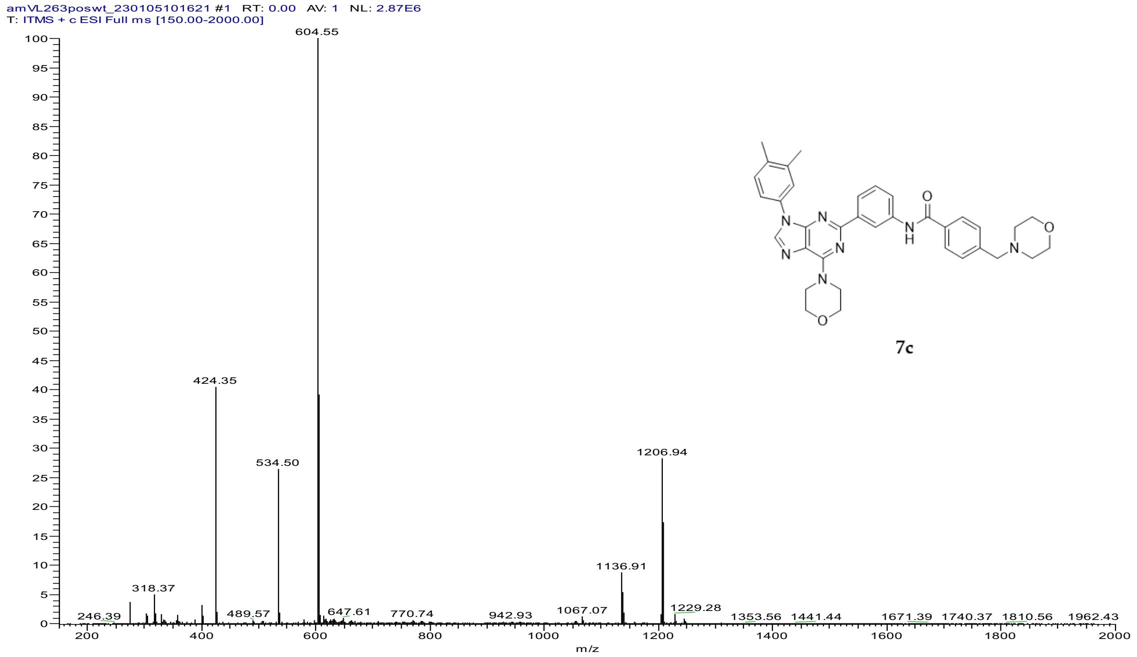


## Compound 7e

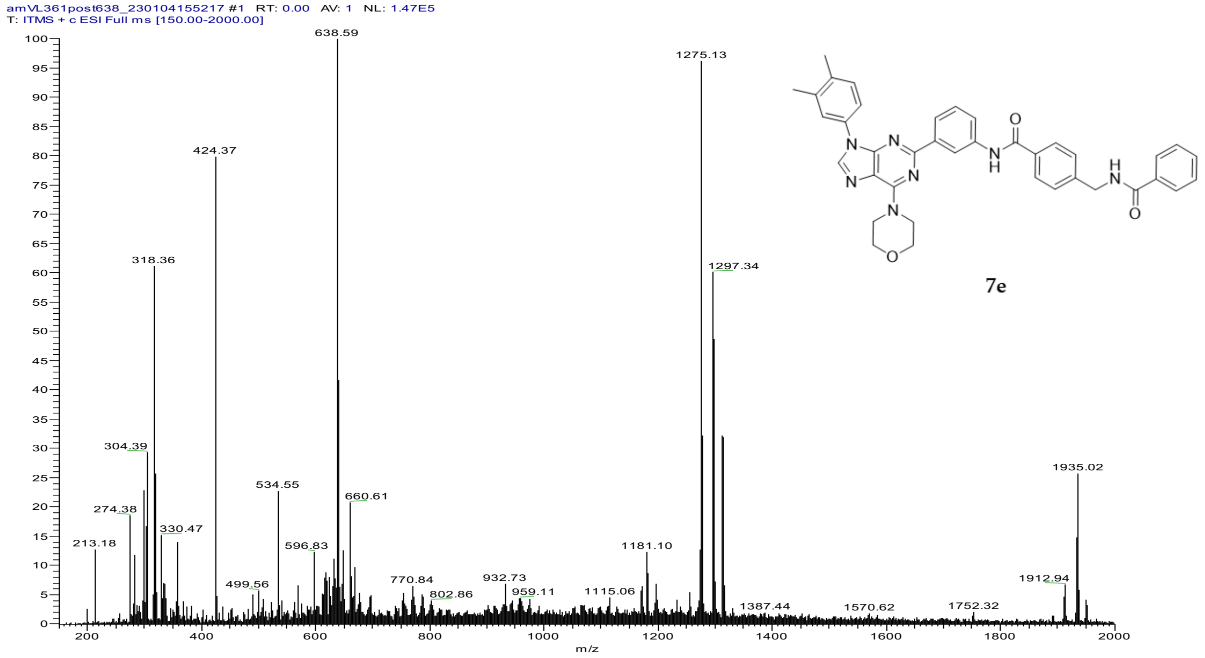

**Table S6.** cont.

## Compound 14b

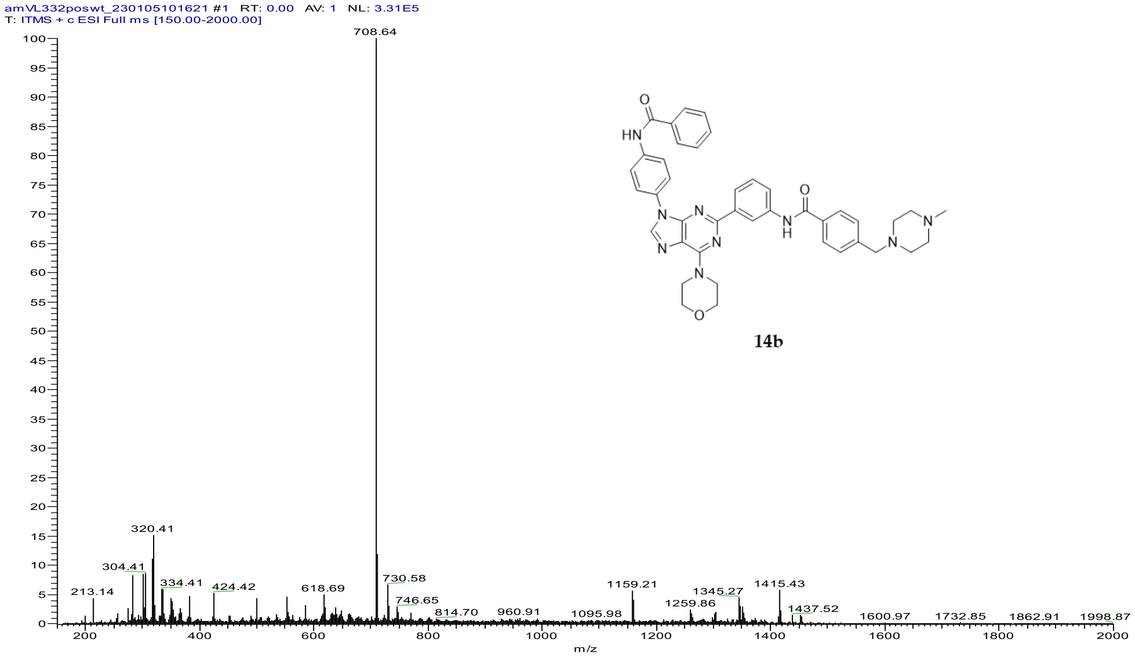

Supplement: Supplementary file 1 [file polymers-15-01703-s001.zip › polymers-2259308-supplementary.pdf]
